# Optical Oxygen Sensors Show Reversible Cross-Talk and/or Degradation in the Presence of Nitrogen Dioxide

**DOI:** 10.1021/acssensors.2c01385

**Published:** 2022-09-16

**Authors:** Irene Dalfen, Arjan Pol, Sergey M. Borisov

**Affiliations:** † Institute of Analytical Chemistry and Food Chemistry, Graz University of Technology, Stremayrgasse 9, 8010 Graz, Austria; ‡ Research Institute for Biological and Environmental Sciences, Department of Microbiology, Radboud University Nijmegen, Heyendaalseweg 135, 6525 AJ Nijmegen, The Netherlands

**Keywords:** luminescence, quenching, indicator, dye, NO_*x*_, optode

## Abstract

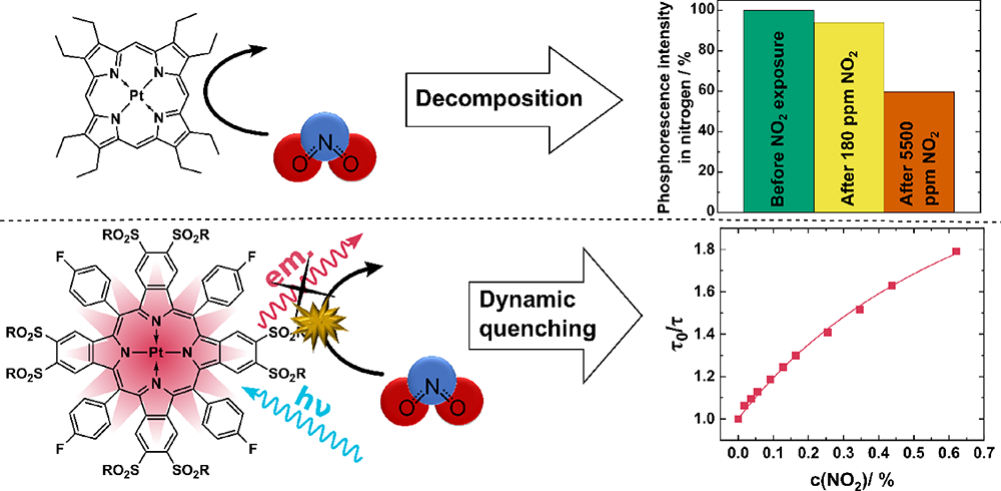

A variety of luminescent dyes including the most common indicators for optical oxygen sensors were investigated in regard to their stability and photophysical properties in the presence of nitrogen dioxide. The dyes were immobilized in polystyrene and subjected to NO_2_ concentrations from 40 to 5500 ppm. The majority of dyes show fast degradation of optical properties due to the reaction with NO_2_. The class of phosphorescent metalloporphyrins shows the highest resistance against nitrogen dioxide. Among them, palladium(II) and platinum(II) complexes of octasubstituted sulfonylated benzoporphyrins are identified as the most stable dyes with almost no decomposition in the presence of NO_2_. The phosphorescence of these dyes is reversibly quenched by nitrogen dioxide. Immobilized in various polymeric matrices, the sulfonylated Pt(II) benzoporphyrin demonstrates about one order of magnitude more efficient quenching by NO_2_ than by molecular oxygen. Our study demonstrates that virtually all commercially available and reported optical oxygen sensors are likely to show either irreversible decomposition in the presence of nitrogen dioxide or reversible luminescence quenching. They should be used with extreme caution if NO_2_ is present in relatively high concentrations or it may be generated from other species such as nitric oxide. As an important consequence of nearly anoxic systems, production of nitrogen dioxide or nitric oxide may be therefore erroneously interpreted as an increase in oxygen concentration.

Optical oxygen sensors (optodes) represent reliable and robust analytical tools with a variety of applications in industry, medicine, biotechnology, environmental monitoring, etc.^1−4^ Commercial versions are available from PreSens, Ocean Optics, PyroScience, Aanderaa, Oxford Optronix, and numerous other producers.^4^ Optodes are advantageous due to a variety of available formats (planar and fiber-optic sensors and nanoparticles) and robustness in terms of interferences, both electromagnetic and chemical. Since optodes rely on dynamic quenching of luminescence by molecular oxygen, chemical interferences are supposed to be rare. Whereas potential cross-talk from hydrophilic species is typically eliminated by immobilization of indicators into hydrophobic matrices, this is not the case for gases that can diffuse into the matrix material and cause interference. To the best of our knowledge, there are only a few literature reports where potential interferences are documented. Particularly, chlorine was shown to quench luminescence of ruthenium(II) polypyridyl complexes immobilized into silicone rubber.^5^ Sulfur dioxide induced strong luminescence quenching of a rhodamine dye immobilized into silicone rubber and organically modified silica, whereas the emission of ruthenium-tris(4,7-diphenyl-1,10-phenanthroline) immobilized in the same matrix was seemingly not affected.^6,7^ Similarly, SO_2_ was reported to very efficiently quench the luminescence of Pd(II) coproporphyrin Langmuir–Blodgett films but had only a small influence on the luminescence of PtOEP in polystyrene.^8^

Nitrogen dioxide is a major air pollutant resulting from combustion of fossil fuels in engines and power plants. For instance, the European parliament has set the upper limit for the annual limit value for the protection of human health to 40 μg m^–3^, which equals 20 ppb.^9^ Nitrogen dioxide can also be formed from nitric oxide, a signaling compound in pro- and eukaryotes. In plants, nitric oxide plays a role in regulating respiratory activity, and in microorganisms, it is an intermediate in nitrification/denitrification pathways that may be encountered in oxic and anoxic environments, respectively, like biofilms or sediments.^10−14^ Although denitrification is conventionally considered to be an anaerobic process, it may still occur at low oxygen concentrations, and so, both NO and oxygen may be present at the same time. This makes reliable simultaneous quantification of NO and oxygen very important.^15,16^ Despite the fact that optodes have become tools of choice to measure oxygen in many research fields, the possible cross-talk of these sensors to NO and NO_2_ has largely not been considered. A recent report indicated minor cross-sensitivity of an oxygen optode to NO, but the potential influence of NO_2_ has not been investigated.^15^

Several literature reports document luminescence quenching by NO_2_ for some chromophores including a ruthenium(II) complex,^5^ metal-free porphyrin,^17^ ZnO nanowires,^18^ and CdSe/ZnS quantum dots^19^ and propose to utilize this process for optical sensing of nitrogen dioxide. Other reported optical sensors for nitrogen dioxide utilize modulation of the energy transfer between linker and metal nodes in a lanthanide metal–organic framework (MOF) leading to luminescence enhancement or quenching,^20^ luminescence quenching in another lanthanide MOF via electron exchange with NO_2_ acting as an electron acceptor,^21^ fluorescence quenching due to disruption of highly emissive J-aggregates in Langmuir–Blodgett films,^22^ or reversible absorption changes upon interactions of NO_2_ with a porphyrin^23^ or osmium(II) tris-bipyridyl complex.^24^ The above literature reports indicate that at least some of the state-of-the-art optical oxygen sensors are likely to show a cross-talk to nitrogen dioxide that may reduce the measurement precision or even completely compromise the measurements under certain conditions.

Therefore, in the current contribution, we investigate the effect of NO_2_ on luminescent properties of common oxygen indicators along with several dyes of other classes that are immobilized into polystyrene to give optical sensing materials. We will show that in the majority of cases, nitrogen dioxide induces irreversible decomposition of the indicator, and even in the case of chemically robust dyes, reversible luminescence quenching is observed.

## Experimental Section

All chemicals used in this study were purchased from commercial suppliers, and unless otherwise indicated, they were used as received. NO_2_ gas was supplied by Linde as NO_2_/N_2_O_4_ with a purity of 99.99% (www.linde-gas.at). A detailed list of chemicals can be found in the Supporting Information. Experiments were conducted at 25 °C with an average ambient pressure of 972 hPa.

### Optical Sensor Foils

**Figure 1:**
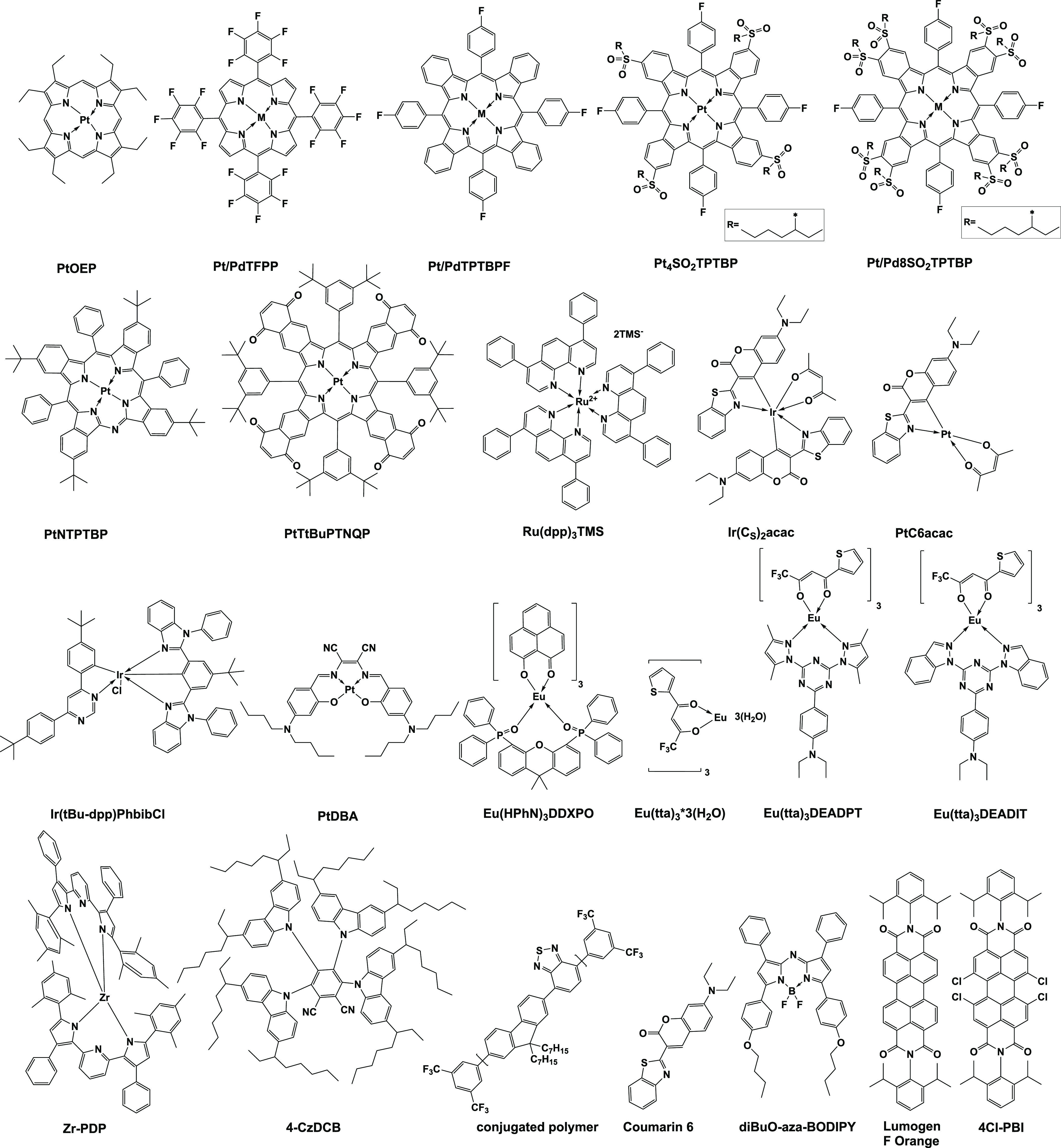
Overview of the investigated dyes and their respective abbreviations.

Chemical structures of the indicator dyes investigated in this study are displayed in Figure 1, and a full list of the indicators including their commercial source or synthesis procedure can be found in the Supporting Information (Table S1).

Foils of the dyes in polystyrene (PS) were produced by knife-coating a “cocktail” of a dye and PS dissolved in either tetrahydrofuran or toluene onto a polyethylene terephthalate (PET) support. Immediately after coating, a 0.45 μm Fluoropore PTFE filter was applied on top of the wet foils so that the “cocktail” would penetrate into the filter’s pores. This was done to provide a signal-enhancing scattering layer and simultaneously to ensure fast dynamic response due to faster gas diffusion in the microstructured polymer layer. Detailed composition of the “cocktails” can be found in the Supporting Information (Table S2).

In addition to PS sensor foils, PtTFPP, PtTPTBPF, and Pt8SO_2_TPTBP were further investigated in other polymeric matrices. In the case of PtTFPP, these were syndiotactic polystyrene (sPS), polysulfone (PSU), poly(2,2,2-trifluoroethylmethacrylate) (pTFEMA), Dyneon Thermoplastic THV221AZ (Dyneon), poly(ethylene chlorotrifluoroethylene) (ECTFE), Cytop CTL-M (Cytop), and poly(aryl ethers) M99, M112, M123, and M127. PtTPTBPF was also investigated in sPS, pTFEMA, PSU, M99–M127, and additionally in poly(pentafluorosytrene) (pPFS) and brominated polystyrene (PS-Br). Finally, Pt8SO_2_TPTBP was immobilized in M99, M127, and pTFEMA.

### Instrumentation

The phosphorescence lifetime and luminescence intensity were determined by phase fluorometry. PtOEP, PtTFPP, PdTFPP, PtT*t*BuTNQP,^25^ Eu(tta)_3_·3H_2_O, Eu(tta)_3_DEADPT,^26^ and Eu(tta)_3_DEADIT^27^ were read out by an SR830 DSP lock-in amplifier from Stanford Research Systems (www.thinksrs.com) equipped with a PMT module (H5701–02) from Hamamatsu (www.sales.hamamatsu.com) and an appropriate set of the LED and excitation and emission filters (see Table S3, SI for more details). Pt(II) and Pd(II) benzoporphyrins, PtNTPTBP,^28^ PtDBA^29^ and diBuO-aza-BODIPY^30^ were characterized using a FireSting-O_2_ module from PyroScience (www.pyroscience.com) that utilizes a 625 nm red LED for the excitation and records the emission in the far-red–NIR part of the spectrum. Ir(C_S_)_2_acac,^31^ Ir(*t*Bu-dpp)PhbibCl,^32^ PtC6acac,^33^ Ru(dpp)_3_TMS_2_, Zr-PDP,^34^ 4-CzDCB (prepared analogously to Steinegger et al.^35^), Eu(HPhN)_3_DDXPO,^36^ poly(9,9-dihepthylfluorene-alt-benzothiadiazole),^37^ coumarin 6, Lumogen F Orange, and 4Cl-PBI were read out using a custom version of the FireSting-Pro module from PyroScience (www.pyroscience.com) equipped with a blue LED. The measurement frequency in all cases was one measurement point every 10 s to minimize possible photobleaching effects.

**Scheme 1:**
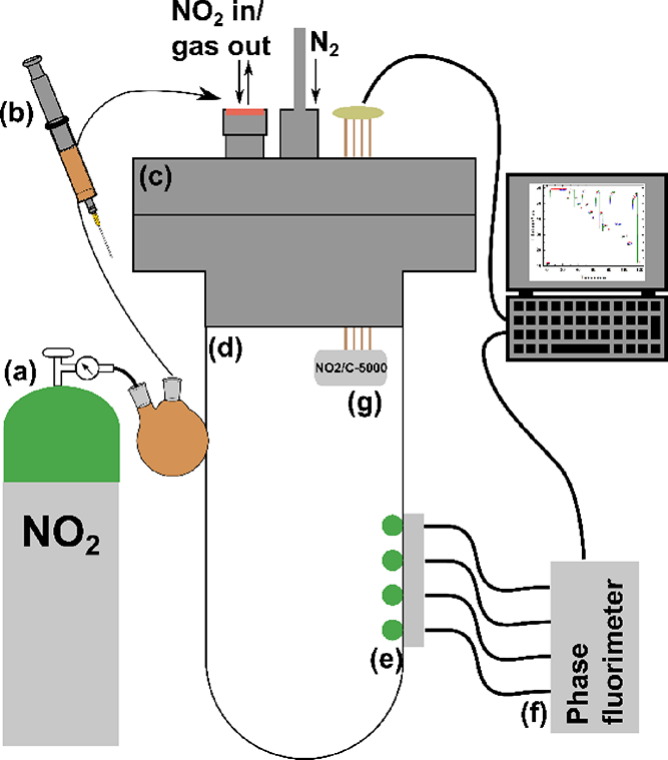
Schematic Representation of the Measurement Setup (Not to Scale) NO_2_ supplied from the gas bottle (a) was first transferred to a round-bottom flask and from there injected with a syringe (b) into the domed glass tube (d). The tube was equipped with a stainless-steel flange lid (c) that contained feedthroughs for nitrogen gas and the electrochemical NO_2_ sensor (g) and a screw cap equipped with a septum for gas insertion that could be removed for purging the setup with nitrogen. The sensor (g) was positioned inside of the glass tube. Dye-doped polymer foils (e) were attached to the inner side of the glass tube and read out with a phase fluorimeter (f).

The measurements were conducted in a custom-made gastight setup (Scheme 1) consisting of a domed end glass tube (*V* = 548 mL) welded to a stainless-steel flange (both from LewVac (www.lewvac.co.uk)). The flange was equipped with gas feedthroughs to attach a nitrogen line for flushing with N_2_ and a screw cap equipped with a septum for injection of NO_2_ with a 1000 μL glass syringe. The screw cap could be removed to allow purging with nitrogen. An electrical feedthrough allowed connection of the electrochemical NO_2_ sensor (NO2/C-5000 from Membrapor, www.membrapor.ch) inside the setup to the readout electronics. The dye foils were attached to the inner wall of the glass tube with a small spot of clear vacuum grease and read out via optical fibers leading to a phase fluorimeter that were attached to the outside of the glass tube with a custom 3D printed fiber holder.

## Results & Discussion

### Choice of Indicators and Polymers

In order to investigate the influence of NO_2_ on optical sensor materials, luminescent dyes that belong to various classes were selected. Phosphorescent dyes represent the largest group since they are either routinely applied in optical oxygen sensors (PtOEP, PtTFPP, PdTFPP, Ru(dpp)_3_^2+^, and PtTPTBPF^1−4,38^) including commercially available sensors or have been reported as promising indicators for some more specific applications (It(Cs)_2_acac,^31^ Eu(HPhN)_3_DDXPO,^36^ PtDBA,^29^ PtNTPTBP,^28^ Pt4SO_2_TPTBP, and Pt/Pd8SO_2_TPTBP^39^). The large group of metalloporphyrins makes it possible to assess the effect of electron-withdrawing substituents (PtOEP-PtTFPP and PtTPTBP-Pt4SO_2_TPTBP-Pt8SO_2_TPTBP), π-extension (porphyrins–tetrabenzoporphyrins–naphthoquinonoporphyrin PtT*t*BuTNQP), core substitution with nitrogen (PtNTPBP), and longer phosphorescence decay times (Pt(II)–Pd(II) complexes). Extremely bright cylcometalated complexes of Ir(III) (Ir(Cs)_2_acac and Ir(*t*Bu-dpp)PhbibCl) and the analogous cylcometalated Pt(II) complex PtC6acac were also included. Finally, two compounds featuring thermally activated delayed fluorescence (TADF) with triplet-state decay times in the microsecond time domain (Zr-PDP^34^ and 4-Cz-DCB, a highly soluble analogue of tetra-(3,6-di-*tert*-butyl-9*H*-carbazole)-1,2-dicyanobenzene published by Steinegger et al.^35^) were chosen. Luminescent Eu(III) complexes generally show relatively long excited-state decay times (hundreds of microseconds) but only little oxygen sensitivity and for this reason are interesting candidates for investigation of the NO_2_ effect on luminescent properties. We selected a simple commercially available β-diketonate complex Eu(tta)_3_·3H_2_O and two representatives equipped with antennas for excitation with visible light (Eu(tta)_3_DEADPT and Eu(tta)_3_DEADIT).^27,40^ Rather uniquely among this class of compounds, the luminescence of another visible-light-excitable complex Eu(HPhN)_3_DDXPO was demonstrated to be efficiently quenched by molecular oxygen.^36^ Finally, several fluorescent dyes (coumarin C6, aza-BODIPY, perylenes, and a conjugated polymer) were added for comparison purposes.

In the first step, all the dyes were investigated as solutions in polystyrene, a very common matrix for preparation of optical oxygen sensors that is characterized by high optical transparency, excellent chemical stability, and good compatibility with lipophilic dyes. To ensure fast response, a porous Teflon filter was soaked with the “cocktail” containing dyes and polymers dissolved in an organic solvent to give readily gas-accessible microstructurized surfaces after solvent evaporation. The additional positive effect is a higher signal-to-noise ratio due to scattering by the white filter. Because Teflon is chemically inert at ambient conditions, these modifications can be assumed to be merely in the physical structure of the sensor film and not altering the chemical behavior of the components.

The same strategy was adapted for other polymers, characterized in combination with two selected indicators PtTFPP and PtTPTBPF. These included partly fluorinated polymers poly(pentafluorostyrene), poly(2,2,2-trifluoromethacrylate (pTFEMA), Dyneon THV221AZ (Dyneon), and poly(ethylene-co-chlorotrifluoroethylene) (ECTFE) that were expected to show higher oxygen permeability and chemical stability compared to the nonfluorinated analogs. Due to compatibility reasons, only fluorinated PtTFPP was used in Dyneon and ECTFE. Additionally, PtTFPP was covalently coupled to amino-modified fumed silica particles that were further dispersed in the amorphous perfluoropolymer Cytop. Other polymers included polysulfone (PSU), brominated polystyrene, and new poly(aryl ethers) M99, M112, M123, and M127. Finally, the most stable indicator, Pt8SO_2_TPTBP, was immobilized in three of these polymers (M99, M127, and pTFEMA) to investigate in detail the quenching of luminescence by NO_2_ and to compare it to quenching by molecular oxygen.

### Stability of Polystyrene-Immobilized Dyes in the Presence of NO_2_

**Figure 2:**
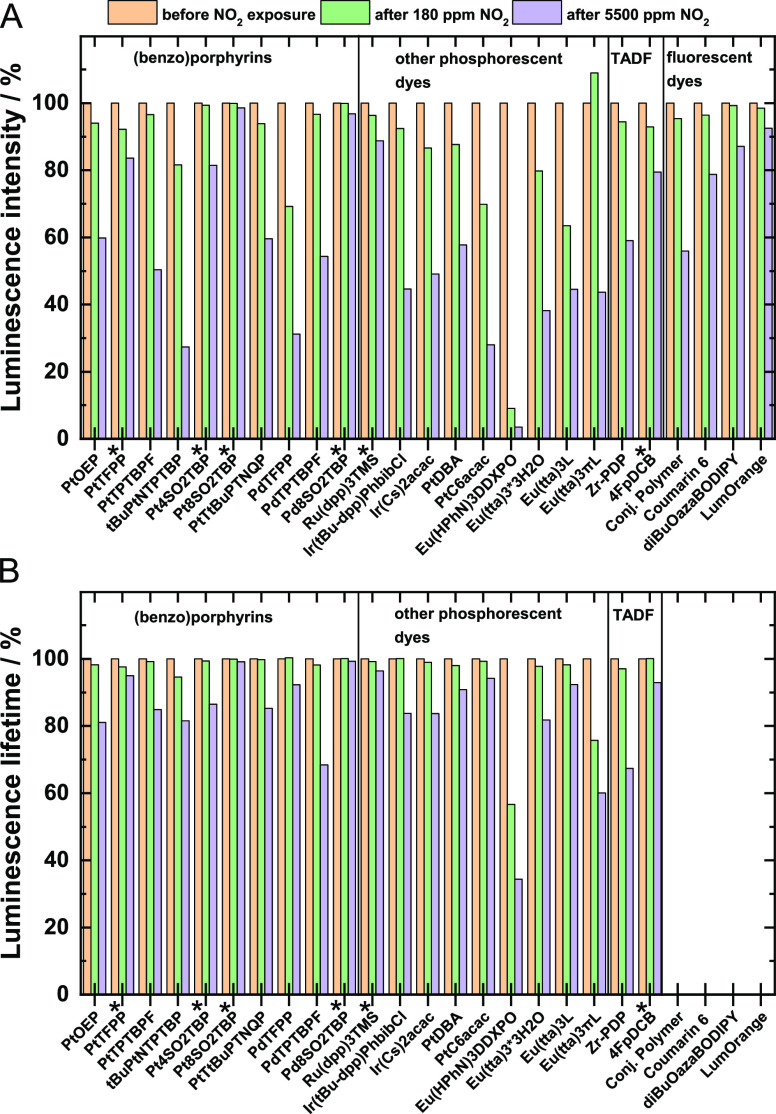
Change of luminescent properties in anoxic conditions (*I*_0_ (A) and τ_0_ (B)) of polystyrene-immobilized dyes upon exposure to ∼180 and ∼5500 ppm NO_2_ for about 15 min. The *I*_0_ and τ_0_ values before exposure to NO_2_ are normalized to 100%. Dyes that show a decrease in luminescence intensity of 20% or less after exposure to 5500 ppm are considered stable enough to quantitatively evaluate their performance and are marked with an asterisk (*).

The stability of the immobilized dyes was accessed via luminescence intensity and decay time measurements (Figure 2) and via UV–vis spectroscopy (Figures S4–S11). The dyes were exposed to ∼180/5500 ppm NO_2_ in nitrogen for about 15 min and then characterized under a nitrogen atmosphere. Most of the dyes were found to show significant degradation of luminescence intensity already after exposure to ∼180 ppm NO_2_ with a much stronger effect of 5500 ppm NO_2_ (Figure 2a). The europium complexes, PdTFPP and PtNTPTBP, were found to be particularly unstable; the majority of dyes occupy the intermediate position; Ru(dpp)_3_TMS, PtTFPP, Pt4SO_2_TPTBP, 4-CzDCB, and fluorescent dyes show fairly good stability, and finally, octasulfonylated complexes Pt8SO_2_TPTBP and Pd8SO_2_TPTBP show almost no degradation even after exposure to comparably high concentrations of nitrogen dioxide.

Numerous biomolecules are known to be degraded by NO_2_ in various mechanisms:^14^ hydrogen abstraction, competing intermolecular addition to double bonds, and electron transfer creating reactive radicals. NO_2_ also is known to react with simple aromatic compounds such as phenols, biphenyls, and polycyclic aromatic compounds by substitution both at the aromatic ring and at aliphatic substituents,^41^ and it is likely that the same type of reaction occurs with the luminescence dyes. In order to obtain more information on degradation pathways, we monitored the absorption spectra and luminescent properties of toluene solutions of several selected dyes (Ir(C_S_)_2_acac, PtTPTBPF, Ru(dpp)_3_(TMS)_2_, and PtOEP) during exposure to an excess of NO_2_. After the experiments, the excess of NO_2_ was removed, and solutions were subjected to analysis via high-resolution mass spectrometry. The results (Figures S12–S22) show formation of numerous products and characteristic changes in optical properties similar to the ones obtained for polystyrene-immobilized dyes. In many cases, the dyes are almost fully converted into various products during the reaction with NO_2_. However, the changes in the absorption spectra often are rather small (cf. Figures S12 and S14 and Figures S18 and S20). Therefore, the chromophore is likely to be preserved in most cases, so the species after the reaction still absorb light in the same part of the electromagnetic spectrum. Correct attribution of formed products is challenging; however, it is evident that the species both with a higher molecular weight and a lower molecular weight are formed in all cases. In the case of Ir(C_S_)_2_acac, the reaction results in addition of NO_2_, elimination of one or two ethyl groups, elimination of the auxiliary ligand acetylacetone, and combined elimination of acetylacetone and one or two ethyl groups (Figure S14). Most if not all of these species are expected to efficiently absorb light similarly to the initial complex. On the other hand, the reactions may strongly affect the luminescent properties. For instance, the luminescence intensity of Ir(C_S_)_2_acac and PtTPTBPF decreases several fold after the reaction with NO_2_ (Figures S13 and S16).

Figures S4–S11 show that in the case of polystyrene-immobilized dyes, changes in absorption spectra are generally similar to the selected dyes investigated in toluene solutions. The absorption spectra do not change significantly for the most stable dyes that showed only minimal degradation in the luminescence intensity. However, it can be noticed that in some cases, the observed changes in the absorption spectra are much less pronounced than in the case of luminescence intensity. In fact, Pt(II) and Pd(II) complexes with *meso*-tetra(pentafluorophenyl)porphyrin TFPP show almost no change in the absorption spectra (Figure S4), whereas the luminescence intensity degrades noticeably, particularly in the case of the Pd(II) complex (Figure 2a). As discussed above, this may be due to a significant difference in luminescent properties but high similarity of the absorption spectra of the reaction products compared to the unmodified porphyrins analogously to what is observed, e.g., for beta-substituted nitro-porphyrins.^42^ It should also be considered that the concentration of the dyes in polystyrene films is rather high (∼1 wt % or ∼10 mM, Table S2) so that such effects as FRET may become considerable since some of the products formed even in small amounts may induce luminescence quenching. Finally, the polymer environment of the dye may be modified resulting in formation of strong luminescence quenchers. Although substitution of tertiary hydrogen atoms in polystyrene by nitro groups is reported to be slow, this possibility cannot be excluded.^43^

Figure 2b shows that for all phosphorescent/TADF-emitting dyes, the emission decay times also decrease after exposure to NO_2_. The relative decrease is, however, significantly smaller than for the luminescence intensity (Figure 2a). Such behavior is not unexpected because complete conversion of a luminescent chromophore into a nonluminescent compound is not expected to affect the measured luminescence decay time since only the remaining initial dye is “visible”.

Drawing general relationships between the chemical structure and stability under NO_2_ exposure is highly speculative due to a great variety of the investigated chromophore structures. However, in the case of metalloporphyrins, some conclusions can be drawn: (i) PtTFPP bearing four strongly electron-withdrawing pentafluorophenyl groups in the *meso* position appears to be much more stable than PtOEP that possesses electron-donating ethyl groups. However, the substitution pattern (*meso*/beta position) of both dyes is different (Figure 1), so the better reactivity of a specific position can be the reason for such behavior; (ii) in the case of π-extended benzoporphyrins, the stability strongly increases from PtTPTBPF to Pt4SO_2_TPTBP and then Pt8SO_2_TPTBP, which along with the analogous Pd(II) complex represents the most stable compound among all investigated dyes. Since the substituent in the *meso* position is the same for all these dyes, the increase in stability can only be explained by (i) an electron-withdrawing effect of alkylsulfone groups (0/4/8) that reduce the reactivity toward strongly electrophilic NO_2_, (ii) steric hindrance via bulky alkylsulfone groups and substitution of potentially reactive positions in the annulated phenyl ring of the porphyrin, and (iii) a combination of both factors.

### Luminescence Quenching of Polystyrene-Immobilized Dyes by NO_2_

**Figure 3:**
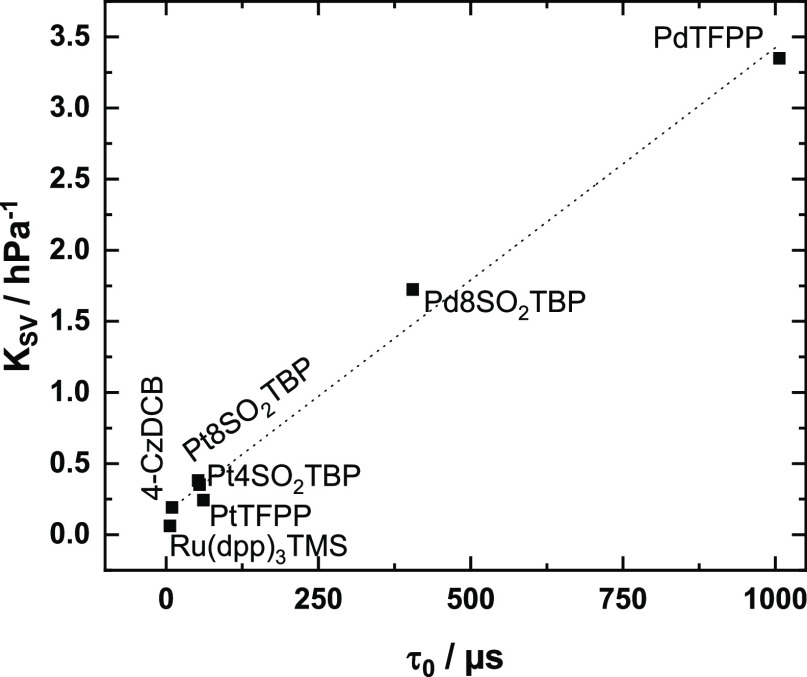
Linear dependence of *K*_SV_ on τ_0_ for the polystyrene-immobilized dyes. *K*_SV_ is calculated from the τ_0_/τ values at ∼180 ppm NO_2_.

Dynamic measurements of luminescence intensity and decay time show that apart from irreversible degradation of photophysical properties, reversible response to NO_2_ is also observed (Figure S23) that is assumed to be due to dynamic luminescence quenching. To assess this component, only dyes with moderate and high stability were selected. In order to further minimize the uncertainty associated with simultaneous quenching and degradation, we assessed the signal change only during exposure to a comparably low NO_2_ concentration of ∼180 ppm. Additionally, the quenching constants were estimated from the luminescence decay times due to a weaker irreversible effect of NO_2_ exposure on this parameter (Figure 2). The quenching constant *K*_SV_ was calculated according to the Stern–Volmer equation:
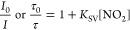
1It should be noted here that the assumption of the linear quenching behavior in polymers represents a very rough approximation. As can be observed from Figure 3, the estimated Stern–Volmer constant is proportional to the luminescence decay time τ_0_, with Ru(dpp)_3_TMS showing the least efficient quenching (τ_0_ = 6.2 μs in PS) and PdTFPP (τ_0_ = 1007 μs in PS) the most efficient. This supports the dynamic quenching mechanism with the bimolecular quenching constant *k*_q_ = *K*_SV_/τ_0_ similar for all the dyes.

### Luminescence Response of Polymer-Immobilized Dyes to NO_2_

**Figure 4:**
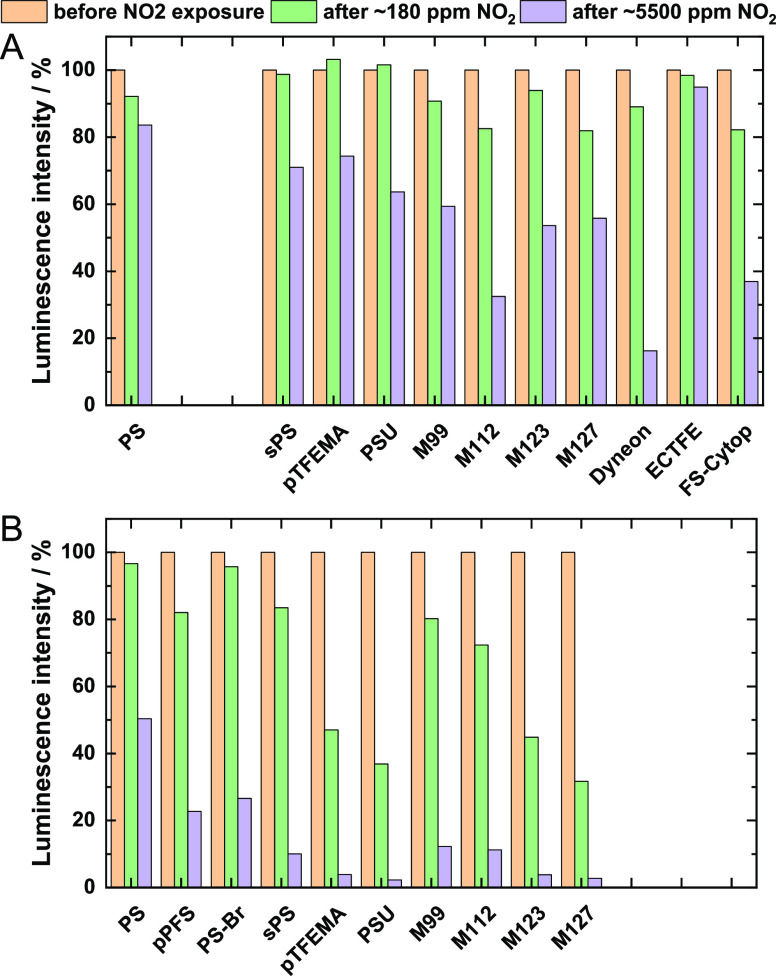
Comparison of normalized luminescence intensity of foils based on PtTFPP (A) and PtTPTBPF (B) in different polymers, before exposure to NO_2_, after exposure to 180 ppm NO_2_, and after exposure to 5500 ppm NO_2_. The time of exposure was about 15 min in all cases.

Since the polymer matrix may considerably affect the stability of dyes in the presence of NO_2_, the alteration of photophysical properties of PtTFPP and PtTPTBPF immobilized into various polymers was investigated. As can be observed from Figure 4 (see Figure S12 for the corresponding τ_0_ values) in most polymers, both dyes show stronger degradation compared to polystyrene materials. It remains unclear if the reaction with NO_2_ is more favored in certain polymers or NO_2_ reacts with polymers generating reactive species/luminescence quenchers that further affect the luminescent properties of the dyes. Poly(ethylene-*co*-chlorotrifluoroethylene) ECTFE represents a notable exception with the highest stability of PtTFPP among all investigated polymers. Unfortunately, partly fluorinated PtTFPP was found to be the only dye compatible with ECTFE.

The τ_0_/τ values at 180 ppm NO_2_ for PtTFPP (that among the two dyes showed superior stability in all the polymers) were used to estimate the *K*_SV_ and *k*_q_ quenching constants (Table 1). As can be seen, the quenching constants vary by several fold that likely reflects different NO_2_ permeabilities of these polymers. The new poly(aryl ether) M127 shows by far the highest sensitivity that is around 3-fold higher than that for the PS-immobilized dye. In contrast, the sensitivity of PtTFPP in other poly(aryl ethers) is much lower. We did not observe noticeable quenching for the ECTFE-immobilized dye.

**Table 1:** Estimated Constants for Luminescence Quenching of Polymer-Immobilized PtTFPP by NO_2_

polymer	*K*_SV_ (hPa^–1^)	τ_0_ (μs)	*k*_q_ (·10^–3^) (hPa^–1^ s^–1^)
PS	0.25	61	4.1
sPS	0.59	72	8.2
pTFEMA	0.48	82	5.9
PSU	0.27	68	4.0
M99	0.28	80	3.5
M112	0.31	82	3.8
M123	0.74	81	9.1
M127	1.03	78	13.2
Dyneon	0.16	69	2.3

It should be mentioned here that PtTFPP has been an extremely popular indicator in optical oxygen sensors including numerous commercial products.^1^ Therefore, an important conclusion of our study is that virtually any of these materials are very likely to show at least some instability in the presence of NO_2_. Prolonged exposure to this gas even in much smaller concentrations than 180 ppm is likely to accelerate the drift of the sensors. Moreover, these materials are also expected to show a reversible cross-talk to nitrogen dioxide. The same refers to the palladium(II) analog PdTFPP that is commonly applied in trace oxygen sensors^15^ and, as shown above (Figure 3), is characterized by the highest cross-talk to NO_2_ due to its fairly long luminescence decay time. In the case of the latter, even considerably small concentrations of NO_2_ that may be produced in biological systems may already cause a significant overestimation of oxygen concentration.

### Quenching of Polymer-Immobilized Pt8SO_2_TPTBP by NO_2_ and O_2_

**Figure 5:**
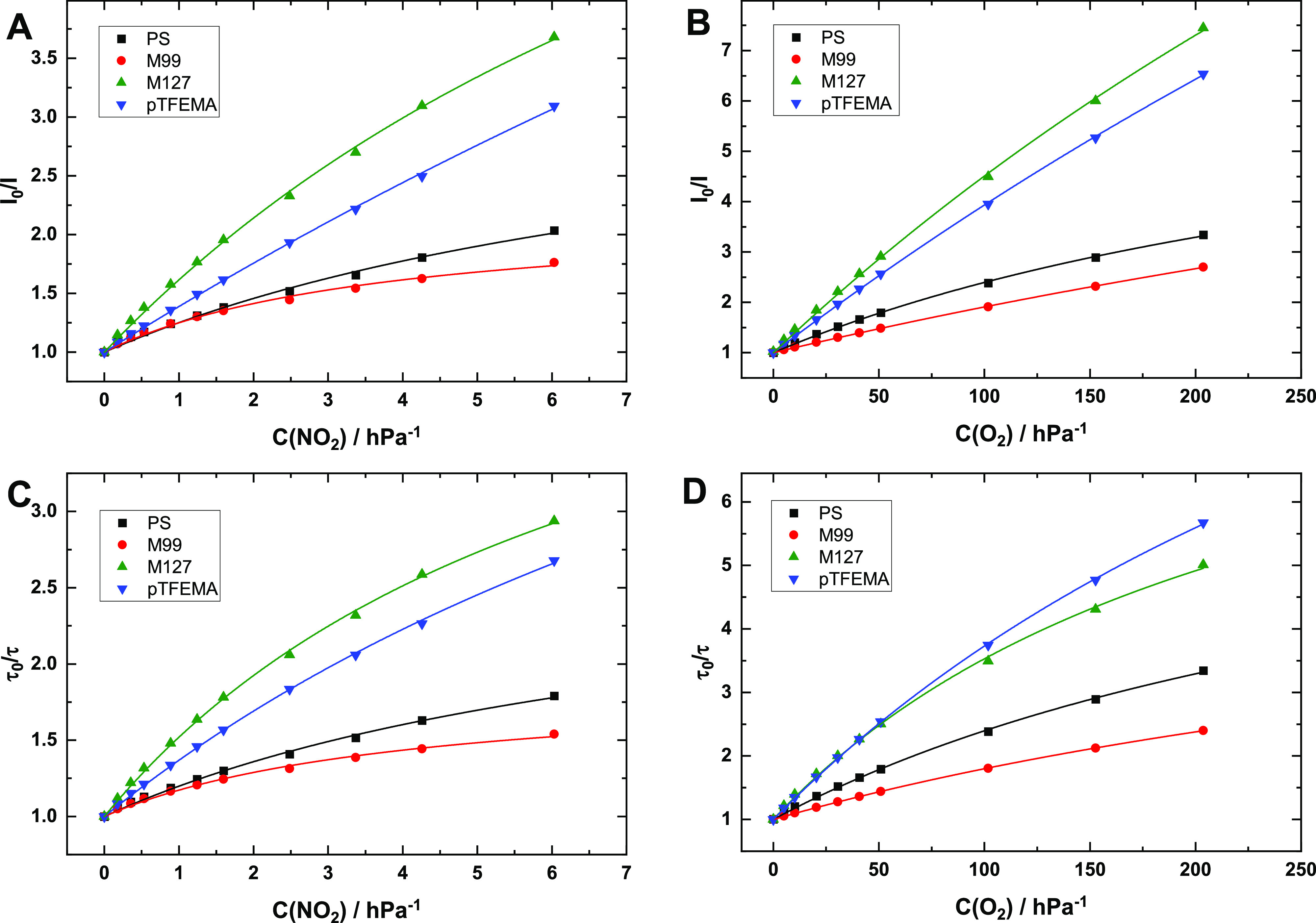
Luminescence quenching of polymer-immobilized Pt8SO_2_TPTBP by NO_2_ (A,C) and O_2_ (B,D). The fit is performed according to eq 3.

In order to investigate quenching of luminescence by NO_2_ in more detail, we selected the octasulfonyl-substituted benzoporphyrin Pt8SO_2_TPTBP. Immobilized in polystyrene, this dye showed the highest stability in the presence of nitrogen dioxide (Figure 2), which makes it possible to neglect decomposition of the dye due to short exposure times. For the detailed studies, we selected PS, pTFEMA, and two new poly(aryl ethers) M99 and M127 that in the screening experiments with PtTFPP showed the lowest and the highest *k*_q_ value, respectively (Table 1). Figure S14 shows that the stability of Pt8SO_2_TPTBP in pTFEMA is comparable to that of PS and is lower in M99 and M127 but nevertheless acceptably high for the detailed investigation. The Stern–Volmer plots for quenching by NO_2_ are summarized in Figure 5A (luminescence intensity) and Figure 5C (luminescence decay time). Figure 5B,D provides the comparison for luminescence quenching by molecular oxygen for the same materials. As can be seen, the quenching behavior for both gases is strikingly similar: (i) the Stern–Volmer plots considerably deviate from linearity, which is known to be very common for oxygen quenching of polymer-immobilized dyes;^44^ (ii) the Stern–Volmer plots for the luminescence intensity and the decay time are generally similar, but the former shows better linearity; and (iii) the efficiency of luminescence quenching by nitrogen dioxide and oxygen generally shows a good correlation for the investigated polymers. M99 shows the lowest sensitivity followed by polystyrene, whereas the sensitivity in M127 is the highest. The strong similarity in quenching behavior for both analytes leads to the conclusion that luminescence quenching by NO_2_ is purely dynamic in nature.

The downward curvature of the Stern–Volmer plots (in contrast to linear plots observed for indicator solutions) is commonly explained by localization of the dye in two microenvironments characterized by significantly different accessibilities to the quencher^45^ (“two-site model” of Demas and coworkers).^46^ Accordingly, the experimental data can be fit with the modified Stern–Volmer equation:
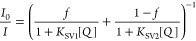
2where *K*_SV1_ and *K*_SV2_ represent the Stern–Volmer constants for the two microenvironments and *f* is the contribution of the first environment. Although physically meaningful only for the luminescence intensity, the equation is also known to excellently fit the decay time plots. In many cases, *K*_SV2_ can be set to 0, which simplifies the equation to the one with only two fit parameters:
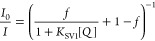
3Indeed, eq 3 fits the Stern–Volmer plots for NO_2_ well (Figure 4 and Table 2). The fit parameters obtained from the luminescence intensity plots are summarized in Table 2, and analogous data from the decay time plots can be found in the Supporting Information (Table S5). First, the obtained *f* values confirm the higher linearity of Stern–Volmer plots for the luminescence intensity compared to the decay time plots (Table S5). Second, the Stern–Volmer plots are found to be more linear for oxygen (higher *f* values) than for NO_2_. This might be due to the comparably good penetration of oxygen in both amorphous and crystalline microenvironments, whereas the solubility or diffusion of highly polar NO_2_ in some of the environments may be reduced. Finally, the most important finding is the significantly more efficient quenching capability of nitrogen dioxide compared to oxygen. In fact, the obtained Stern–Volmer constants *K*_SV1_ for NO_2_ and O_2_ differ by more than one order of magnitude (Table 2). Such a huge difference may be mostly due to a different quenching mechanism. Apart from some cyclometalated complexes,^47^ quenching of phosphorescent dyes by molecular oxygen occurs exclusively via triplet–triplet energy transfer from the emitter to O_2_. Due to the contribution of the spin statistical factor the bimolecular constant, *k*_q_ in this case can only reach 1/9 of the diffusion constant *k*_diff_.^48^ In contrast to molecular oxygen, NO_2_ is a doublet, which would implement that triplet–doublet energy transfer takes place.^49^ Due to the highly electrophilic nature of nitrogen dioxide, quenching via electron transfer also appears to be possible. The better permeability of NO_2_ in some polymers may be an additional contributing factor. For instance, despite the lower diffusion coefficient of NO_2_ in polytetrafluoroethylene, its solubility is significantly higher than that for O_2_, which results in an overall higher gas permeability.^50^ Unfortunately, in contrast to the wealth of data on permeability of oxygen and other gases in polymers, information on NO_2_ permeability is extremely scarce.^51^

**Table 2:** Fit Parameters for the Intensity-Based Stern–Volmer Plots (Fit with Equation 3) Obtained for Immobilized Pt8SO_2_TPTBP

	quencher					
	NO_2_			O_2_		
polymer	*K*_SV1_, hPa^–1^	*f*	*R*^2^	*K*_SV1_, hPa^–1^	*f*	*R*^2^
PS	0.39	0.72	0.996	0.021	0.86	0.999
M99	0.58	0.55	0.993	0.011	0.91	0.999
pTFEMA	0.42	0.94	0.999	0.033	0.97	0.999
M127	0.75	0.89	0.999	0.041	0.97	0.999

## Conclusions

We have shown that the emission of all investigated phosphorescent dyes is affected by the presence of NO_2_. In most cases, NO_2_ causes irreversible degradation of the dyes, which was documented for the polystyrene matrix. A more detailed study with selected dyes and a variety of polymers indicates that almost any dye–polymer combination is likely to be prone to degradation. Complexes of octasulfonylated benzoporphyrins represent a notable exception, but even for this class of phosphorescent emitters, NO_2_ acts as a powerful quencher with quenching constants exceeding those known for molecular oxygen by more than one order of magnitude.

The investigated compounds cover the most important classes of phosphorescent indicators routinely applied in optical oxygen sensors including numerous commercial products. Considering that the concentration of NO_2_ even in polluted air is rather low^9^ and in all cases is likely to stay well below 1 ppm, the deteriorating/quenching effect of this gas is likely to be irrelevant for most applications of optical oxygen sensors. However, extreme care should be taken when applying optodes in environments with a higher concentration of NO_2_. In this case, the optodes may show strongly accelerated drift interpreted as an increase of oxygen concentration. Studies in biological systems that generate nitric oxide may be compromised as well since (unnoticed) generation of NO and subsequent conversion of this gas to NO_2_ can be falsely interpreted as production of oxygen. This calls for re-evaluation of some early studies.^16^ The adverse effect of NO_2_ (and indirectly NO) is expected to be even more severe in hypoxic systems. Here, the assumed drastic increase of O_2_ concentration from the overall low basic level may be caused by the generation of small amounts of NO_2_ and subsequent luminescence quenching/degradation of the oxygen indicator.

## Supporting Information Available

Extended descriptions of materials and procedures used in this work and additional data on stability of the investigated materials (PDF)
