# Supplementary material for: Optical Oxygen Sensors Show Reversible Cross-Talk and/or Degradation in the Presence of Nitrogen Dioxide
Source: ACS Sens. 2022 Sep 16;7(10):3057–66. doi: 10.1021/acssensors.2c01385 (PMC9623579; doi:10.1021/acssensors.2c01385)
Supplement: Supplementary file 1 — se2c01385_si_001.pdf [file se2c01385_si_001.pdf]

Supplementary Information

Optical oxygen sensors show reversible cross-talk and/or degradation in presence of nitrogen dioxide

Irene Dalfen[1], Arjan Pol[2], Sergey M. Borisov[1*]

[1] Institute of Analytical Chemistry and Food Chemistry, Graz University of Technology, Stremayrgasse 9, 8010 Graz, Austria
[2] Research Institute for Biological and Environmental Sciences, Department of Microbiology, Radboud University Nijmegen, Heyendaalseweg 135, 6525 AJ Nijmegen, The Netherlands

Experimental section

Materials and Methods

All chemicals were purchased from commercial suppliers. Unless otherwise indicated, chemicals were used as received. Suppliers of indicator dyes include Frontier scientific (www.frontiersci.com/), Acros Organics (now Thermo Fisher Scientific, www.thermofisher.com), Sigma Aldrich (www.sigmaaldrich.com) and Kremer Pigmente (www.kremer-pigmente.com/). Details are listed in *Table S1*.

Fluoropore™ filters (0.45 µM unlaminated PTFE Filter) were from Merck (www.merckmillipore.com), polystyrene (MW 260,000 Da) from Acros Organics (now Thermo Fisher Scientific, www.thermofisher.com), pTFEMA (MW 100,000 Da) and syndiotactic polystyrene (MW 300,000 Da) from Scientific polymer (www.scipoly.com), PSU was from Polysciences (www.polysciences.com). 3M™Dyneon Thermoplastic THV221AZ was a friendly gift from 3M (www.3mdeutschland.de), Halar® 6014 ECTFE (poly(ethylene-co-chlorotrifluoroethylene)) was generously provided by Solvay (www.solvay.com). CTL-107MK (7% wt. Cytop) was bought from AGC (www.agc.com/en/), UV/VIS grade tetrahydrofuran (THF) and toluene were from VWR (www.VWR.com), Purosolve 75/00 from Puretecs (www.puretecs.de) and fumed silica was from Sigma Aldrich (www.sigmaaldrich.com), Poly(ethyleneterephthalate) support foils Melinex 505 were aquired from Pütz (www.puetz-folien.com).

Poly(2,3,4,5,6-pentafluorostyrene) was synthesized according to literature.[1]

Optical glass filters for use in combination with the lock-in amplifier were from Schott (www.schott.com) or Reichmann Feinoptik GmbH (www.reichmenn-feinoptik.de), LEDs were from Roithner (www.roithner-laser.com).

SEC chromatographic analysis was performed on a WGE Dr. Bures SEC3010 (www.wge-dr-bures.de) with THF as eluent (1 mL min−1) and refractive index (RI) detection. Poly(styrene) standards were used for calibration.

IR spectra were taken on an ALPHA-P FT-IR spectrometer from Bruker (www.bruker.com) with a diamond-based attenuated total reflection (ATR) module.

Dyes

The indicator dyes were either purchased from commercial suppliers or synthesized according to literature procedures, the details are summarized in *Table S1.*

Table S1. Commercial supplier or synthesis procedure for investigated dyes

Dye	Abbreviation	Reference or commercial supplier
Platinum(II)-2,3,7,8,12,13,17,18-octaethylporphyrin	PtOEP	Frontier Scientific
Platinum(II)-5,10,15,20-tetrakis-(2,3,4,5,6-pentafluorphenyl)-porphyrin	PtTFPP	Frontier Scientific
Palladium(II)-5,10,15,20-tetrakis-(2,3,4,5,6-pentafluorphenyl)-porphyrin	PdTFPP	Frontier Scientific
Platinum(II)-meso-tetra-4-fluorophenyltetrabenzoporphyrin	PtTPTBPF	Hutter et al. 2014[2]
Palladium(II)-meso-tetra-4-fluorophenyltetrabenzoporphyrin	PdTPTBPF	Hutter et al. 2014[2]
Platinum(II)-5-aza-10,15,20-triphenyltetra(4-*tert*-butyl)benzoporphyrin	PtNTPTBP	Borisov et al. 2010[3]
Platinum(II) meso-tetra(4-fluorophenyl)-tetra(4-(2-ethylhexyl)sulfoneyl))benzoporphyrin	Pt4SO$_2$TPTBP	Zach et al. 2017[4]
Platinum(II) meso-tetra(4-fluorophenyl)-tetra(4,5-bis(2-ethylhexylsulfoneyl))benzoporphyrin	Pt8SO$_2$TPTBP	Zach et al. 2017[4]
Palladium(II) meso-tetra(4-fl uorophenyl)-tetra(4,5-bis(2-ethylhexylsulfoneyl))benzoporphyrin	Pd8SO$_2$TPTBP	Zach et al. 2017[4]
Platinum(II)-meso-tetra-(3,5-di-*tert*-butylphenyl)tetranaphthoquinonoporphyrin	PtT*t*BuPTNQP	Banala et al. 2016[5]
Iridium(III) bis-(3-(benzothiazol-2-yl)-7-(diethylamino)-coumarin) acetylacetonate	Ir(C$_S$)$_2$acac	Borisov et al. 2007[6]
Iridium(II) [4,6-di(4-tert-butylphenyl)pyrimidine 2-[3-tert-butyl-5-(1-phenyl-1H-1,3-benzodiazol-2-yl)phenyl]-1-phenyl-1H-1,3-benzodiazole] chloride	Ir(*t*Bu-dpp)PhbibCl	Shafikov et al. 2019[7]
Platinum(II) 3-(benzothiazol-2-yl)-7-(diethylamino)-coumarin acetylacetonate	PtC6acac	Borisov et al. 2009[8]
Platinum(II) 2,3-bis[(4-dibutylamino-2-hydroxybenzylidene)amino]but-2-enedinitrile	PtDBA	Borisov et al. 2013[9]
Ruthenium (II) tris(4 7-diphenyl-1 10-phenanthroline) trimethylsilylpropansulfonate	Ru(dpp)$_3$TMS$_2$	Klimant et al. 1995[10]
Zirconium(II) 2,6-bis(5-mesitylenyl-3-phenyl-1H-pyrrol-2-yl)pyridine	Zr-PDP	Zhang et al. 2020[11]
Tetra-(3,6-(2-ethylhexyl)-9*H*-carbazole)-1,2-dicyanobenzene	4CzDCB	Steinegger et al. 2017[12]
Europium(III) tris-[9-(hydroxy-ϰO)-1H-phenaleno-1-onatoϰO]-1,1'-(9,9-dimethyl-9H-xanthene-4,5-diyl)bis-1,1-diphenyl-phosphine oxide	Eu(HPhN)$_3$DDXPO	Borisov et al. 2014[13]
Europium(III) tris-thenoyltrifluoro acetonat trihydrate	Eu(tta)$_3$·3H$_2$O	Acros Organics
Europium(III) tris-thenoyltrifluoro acetonat 4-(4,6-di(3,5-dimethyl-1H-pyrazol-1-yl)-1,3,5-triazin-2-yl)-*N,N* -diethylbenzenamine	Eu(tta)$_3$DEADPT	Yang et al. 2004[14]
Europium(III) tris-thenoyltrifluoro acetonat 4-(4,6-bis(1H-indazol-1-yl)-1,3,5-triazin-2-yl)-*N,N*-diethylbenzenamine	Eu(tta)$_3$DEADIT	Borisov et al. 2008[15]
Poly(9,9-diheptylfluorene-alt-(2,1,3)-benzothiadiazole)	Conjugated polymer	Herguth et al. 2002[16]
3-(2’-benzothiazolyl)-7-diethylaminocoumarin	Coumarin 6	Aldrich
BF$_2$ chelate of [5-(4-buthoxyphenyl)-3-phenyl-1H-pyrrol-2-yl][5-(4-buthoxyphenyl)-3-phenylpyrrol-2-ylidene]-amine	diBuO-aza-BODIPY	Strobl et al. 2015[17]
N,N'-bis(2,6-diisopropylphenyl)perylene-3,4,9,10-tetracarboxydiimide	Lumogen F Orange	Kremer Pigmente
1,6,7,12-Tetrachloro-*N,N'*-bis(2,6-diisopropylphenyl)perylene-3,4,9,10-tetracarboxydiimide	4Cl-PBI	Seybold et al. 1988[18]

Immobilization of dyes in polymers

All foils of polystyrene-immobilized dyes were produced in a similar fashion. First, a sensor "cocktail" was prepared containing the respective dye and the polymer dissolved in a suitable solvent with a concentration of 10% wt. of polymer in the solvent. This "cocktail" was stirred for several hours until all the componets were fully dissolved. Then ~200 µL were pipetted onto a PET support foil and knife coated with a wet film thickness of 76.2 µm. Immediately after knife coating, a Fluoropore™ Teflon filter was carefully placed on top of the still wet sensor foil so that the cocktail would soak into the filter pores. The foils were then left to dry at room temperature for several hours.

Table S2 summarizes the concentrations of dyes in PS and the type of solvent used for the PS-based foils. For PtTFPP, PtTPTBPF and Pt8SO_2TPTBP in other polymers, the indicator concentrations in polymers were 1.3 % wt. for PtTFPP and 1.5 % wt. for PtTPTBPF and Pt8SO_2TPTBP. THF was used to as a solvent in "cocktails" based on pPFS, PSU, pTFEMA and Dyneon. Toluene was used for "cocktails" based on PS-Br and poly(aryl ethers).

Table S2. Compositions of PS-based "cocktails"

Indicator	Weight % of indicator with respect to PS	solvent
PtOEP	1.3	Toluene
PtTFPP	1.3	Toluene
PdTFPP	1.3	THF
PtTPTBPF	1.5	Toluene
PdTPTBPF	1.5	Toluene
PtNTBP	1.1	Toluene
Pt4SO_2TPTBP	1.5	Toluene
Pt8SO_2TPTBP	1.5	Toluene
Pd8SO_2TPTBP	1.5	Toluene
PtT*t*BuPTNQP	0.6	Toluene
Ir(C_S)$_2$acac	1.5	THF
Ir(*t*Bu-dpp)PhbibCl	1	THF
PtC6acac	1	THF
PtDBA	1	Toluene
Ru(dpp)$_3$TMS$_2$	1.2	THF
Zr-PDP	1.5	Toluene
4CzDCB	1	Toluene
Eu(HPhN)$_3$DDXPO	1.5	THF
Eu(tta)$_3$·3H_2O	1.5	THF
Eu(tta)$_3$DEADPT	1.5	Toluene
Eu(tta)$_3$DEADIT	1.5	Toluene
Conjugated polymer	10	Toluene
Coumarin 6	1	THF
diBuO-aza-BODIPY	1	Toluene
Lumogen F Orange	0.5	THF
4Cl-PBI	0.5	THF

The read-out of fluorescence and phosphorescence response of the dyes was performed with either a FireSting®-O_2 employing RedFlash tehcnology , a custom model FireSting®-Pro equipped with a blue excitation source, both from PyroScience (www.pyroscience.com) or a lock-in amplifier from Stanford Research Systems (www.thinksrs.com) equipped with a PMT module (H5701–02) from Hamamatsu, (www.sales.hamamatsu.com) with LEDs and filters chosen to match the respective dye's spectral properties. The modulation frequency was tuned depending on the unquenched lifetime of the dyes as well. Table S3 gives an overview which phase fluorometer and modulation frequency and, in the case of lock-in amplifier, what LED and filters were used.

Table S3. Settings for phase-fluorometric measurements

Indicator	Phase fluorometer	LED (peak wavelength)	Excitation filter	Emission filter	Modulation frequency
PtOEP	Lock-in amplifier	Blue (413 nm)	BG12	OG570	4000 Hz
PtTFPP	Lock-in amplifier	Blue (413 nm)	BG12	OG570	4000 Hz
PdTFPP	Lock-in amplifier	Blue (413 nm)	BG12	OG570	153 Hz
PtTPTBPF	FireSting®-O_2				4000 Hz
PdTPTBPF	FireSting®-O_2				400 Hz
PtNTBP	FireSting®-O_2				4000 Hz
Pt4SO_2TPTBP	FireSting®-O_2				4000 Hz
Pt8SO_2TPTBP	FireSting®-O_2				4000 Hz
Pd8SO_2TPTBP	FireSting®-O_2				400 Hz
PtT*t*BuPTNQP	Lock-in amplifier	Green (501 nm)	BG36	OG630	4000 Hz
Ir(C_S)$_2$acac	FireSting®-Pro				20 kHz
Ir(*t*Bu-dpp)PhbibCl	FireSting®-Pro				32 kHz
PtC6acac	FireSting®-Pro				4000 Hz
PtDBA	FireSting®-O_2				4000 Hz
Ru(dpp)$_3$TMS_2	FireSting®-Pro				10 kHz
Zr-PDP	FireSting®-Pro				800 Hz
4CzDCB	FireSting®-Pro				10 kHz
Eu(HPhN)$_3$DDXPO	FireSting®-Pro				2000 Hz
Eu(tta)$_3$·3H_2O	Lock-in amplifier	UV (355 nm)	UG1	OG570	400 Hz
Eu(tta)$_3$DEADPT	Lock-in amplifier	Blue (413 nm)	BG12	OG570	500 Hz
Eu(tta)$_3$DEADIT	Lock-in amplifier	Blue (413 nm)	BG12	OG570	500 Hz
Conjugated polymer	FireSting®-Pro				4000 Hz
Coumarin 6	FireSting®-Pro				4000 Hz
diBuO-aza-BODIPY	FireSting®-O_2				4000 Hz
Lumogen F Orange	FireSting®-Pro				4000 Hz
4Cl-PBI	FireSting®-Pro				4000 Hz

PtTFPP and PtTPTBP in sPS

sPS was pre-dissolved in $CHCl_3$ by heating to 150 °C for 10 minutes in a Monowave 50 synthesis reactor by Anton Paar (www.anton-paar.com). After cooling to room temperature the solution remained stable for several hours. The concentration of PtTFPP was 1.3% wt. with respect to sPS and of

PtTPTBPF - 1.5% wt. with respect to sPS. The respective amount of dye was added to the polymer solution, homogenized by stirring vigorously for ca. 15 minutes and then knife-coated on a PET support with a wet film thickness of 76.2 µm. No Teflon filter was added as the fast evaporation of $CHCl_3$ would not allow for the "cocktail" to soak into the pores.

PtTFPP on fumed silica in Cytop (FS-Cytop)

One sensor foil was produced with PtTFPP coupled to fumed silica and dispersed in Cytop CTL-107MK. The coupling process was conducted according to literature procedure.[19] 50 mg of the modified particles were then dispersed in a "cocktail" containing 720 mg of CTL-107MK and 780 mg Purosolve 75/00. The "cocktail" was stirred for several hours until homogenous distribution was achieved and then knife-coated on an SiO_x-modified PET foil.

PtTFPP in ECTFE

1.5 mg PtTFPP and 99.8 mg ECTFE were added to a 2.5 mL glass vial and 910 mg 1,3-dichlorobenzene were added. The mixture was heated in a stainless steel heating block to 170 °C until ECTFE dissolved. Glass discs with one roughened side (diameter 8 mm, hight 1 mm) were used as a transparent support instead of PET due to poorer stability of the latter at high temperatures. The glass discs also were placed on the heating block and a small portion of "cocktail" was homogenously applied to the roughened side of the hot glass disc using a heated glass pipette. Then the discs were left on the hot heating block for another 15 minutes to evaporate the solvent, after which the heating was turned off to let the discs slowly cool to room temperature.

Poly(aryl ether) polymers

The structures of the synthesized poly(aryl ethers) are shown in *Figure S1*, their synthesis was conducted analogously to a procedure published by Liu et al. in 2003.[20] *Table S4* shows results of SEC chromatography of the synthesized poly(aryl ether) polymers, *Figure S2* contains the IR spectra.

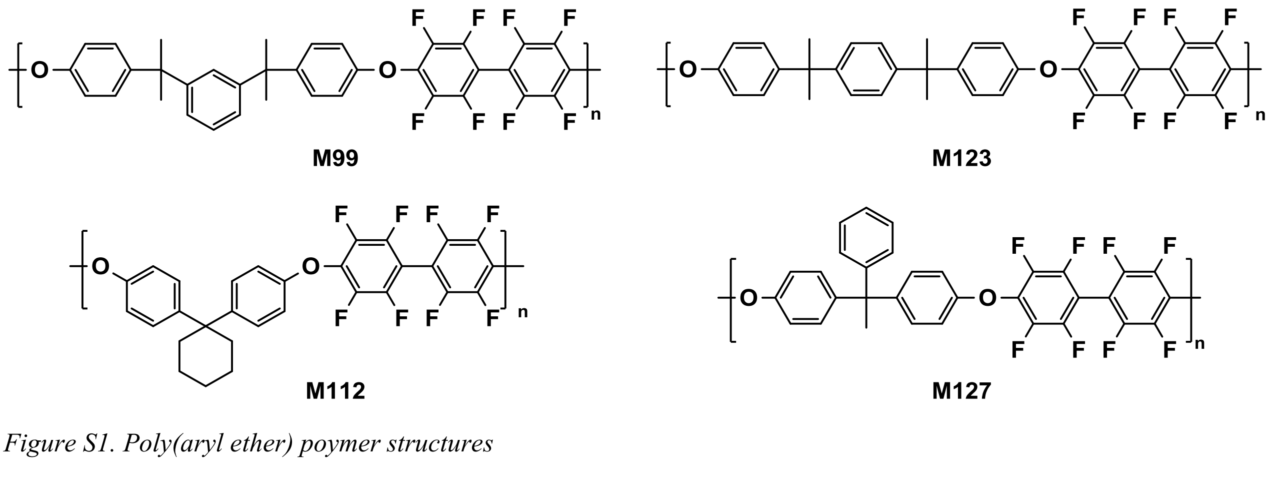

Figure S1. Poly(aryl ether) poymer structures

Table S4. Results of GPC analysis of poly(aryl ether) polymers

	Mn / g mol^{-1}	Mw / g mol^{-1}	PDI
M99	4859	11034	2.27
M112	10200	16604	1.63
M123	8120	12968	1.60
M127	12975	21887	1.69

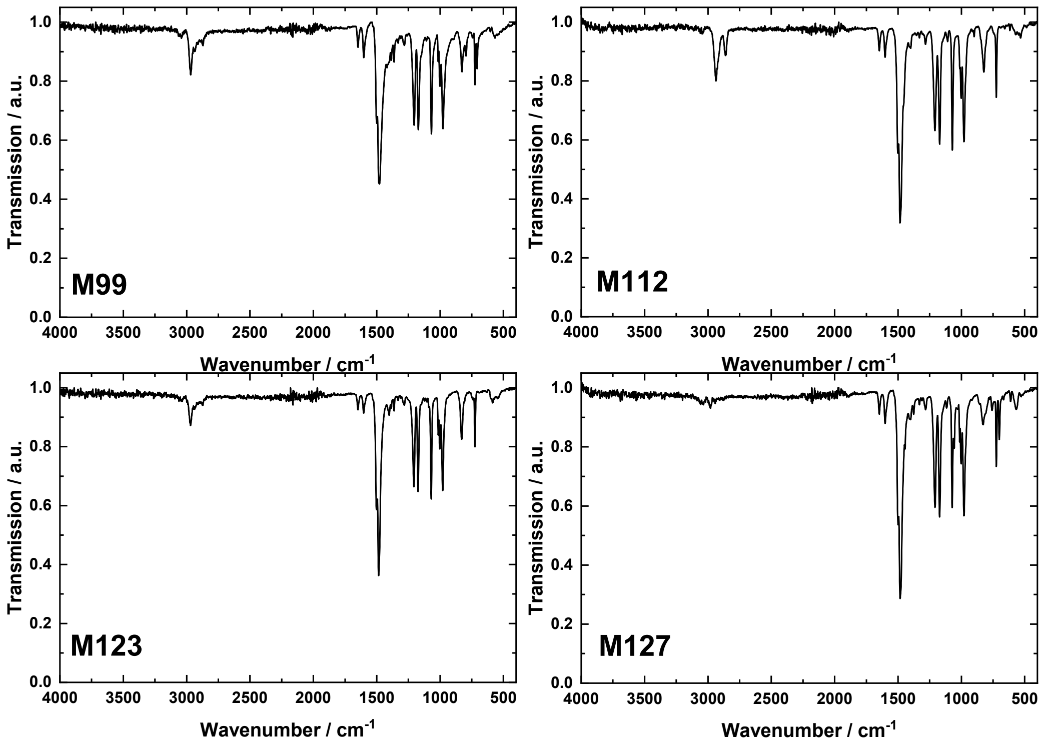

Figure S2. ATR-IR spectra of poly(aryl ether) polymers

Results

An example of a typical measurement is provided in *Figure S3* for two dyes, Pt8SO_2TPTBP and PtTPTBPF, both in PS.

Degradation of dyes due to exposure to small (180 ppm) and high (5500 ppm) concentrations of NO_2 (about 15 minutes exposure time) was determined by comparing the lifetime and intensity of luminescence of PS-based sensor foils in N_2 before any exposure to NO_2 as well as after exposure to the respective concentrations of NO_2. The same was done with materials based on PtTFPP, PtTPTBPF and Pt8SO_2TPTBP in different polymers. The results are depicted in *Figure 2*.

Additionally, absorption spectra of PS-based foils were recorded before exposure to NO_2, after exposure to 180 ppm NO_2 over a duration of 10 minutes and after exposure to 5500 ppm NO_2 over a duration of 30 minutes. In these cases, exposure to NO_2 too place in darkness so no additional photodegradation took place. The results can be seen in *Figures S4-S11*.

Changes in absorption spectra and luminescent properties of selected dyes (Ir(C_S)$_2$acac, PtTPTBPF, Ru(dpp)$_3$(TMS)$_2$ and PtOEP) dissolved in toluene upon exposure to NO_2 as well as mass spectroscopic analysis of resulting products are summarized in *Figures S12-S22.*

The stability of materials based on PtTFPP, PtTPTBPF and Pt8SO$_2$TPTBP in different polymers towards NO_2 was determined as for the PS-based sensors, the results are depicted in *Figures S23 & S24* and in *Figure 4.*

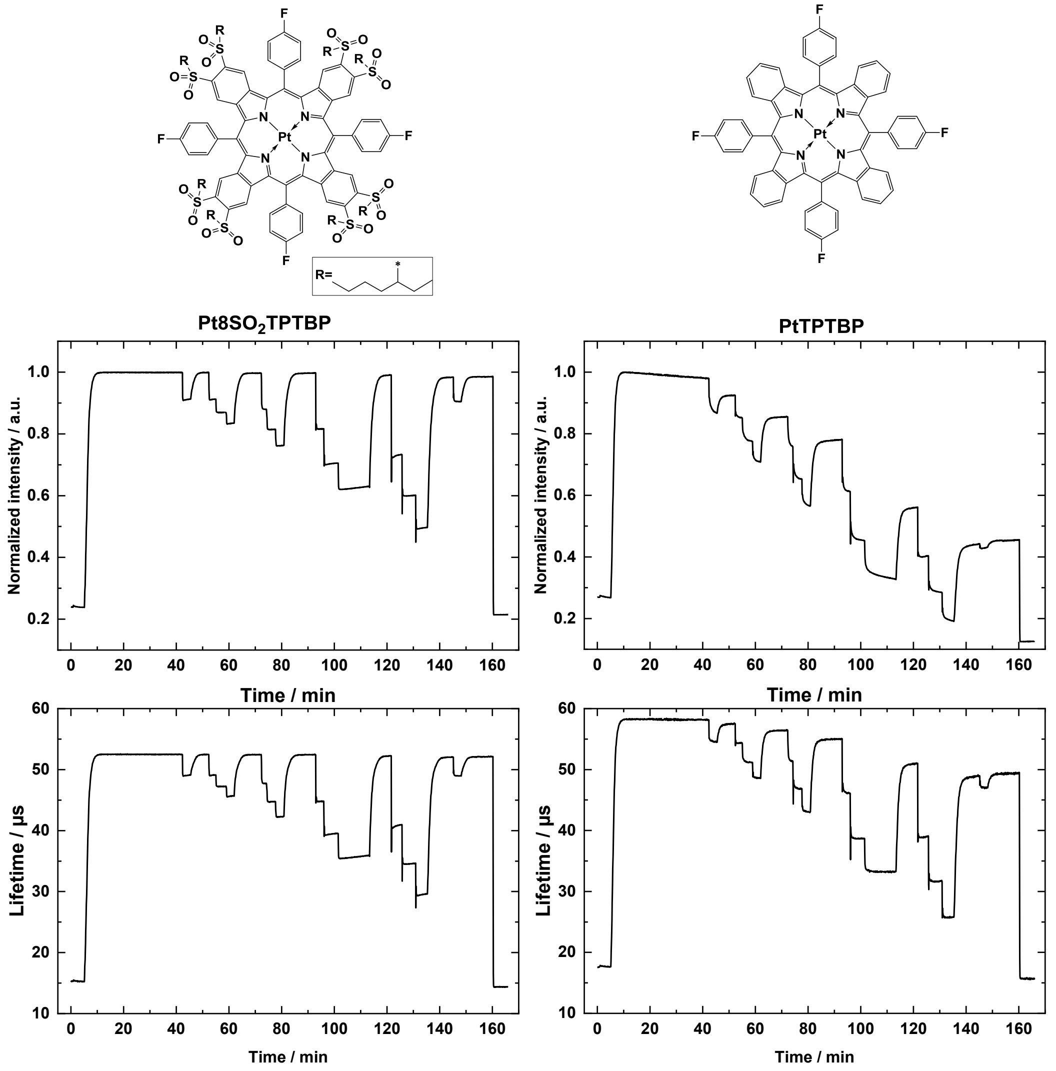

Figure S3. Measurement of luminescence response to NO_2 exposure. Left: Pt8SO$_2$TPTBP in PS, Right: PtTPTBPF in PS

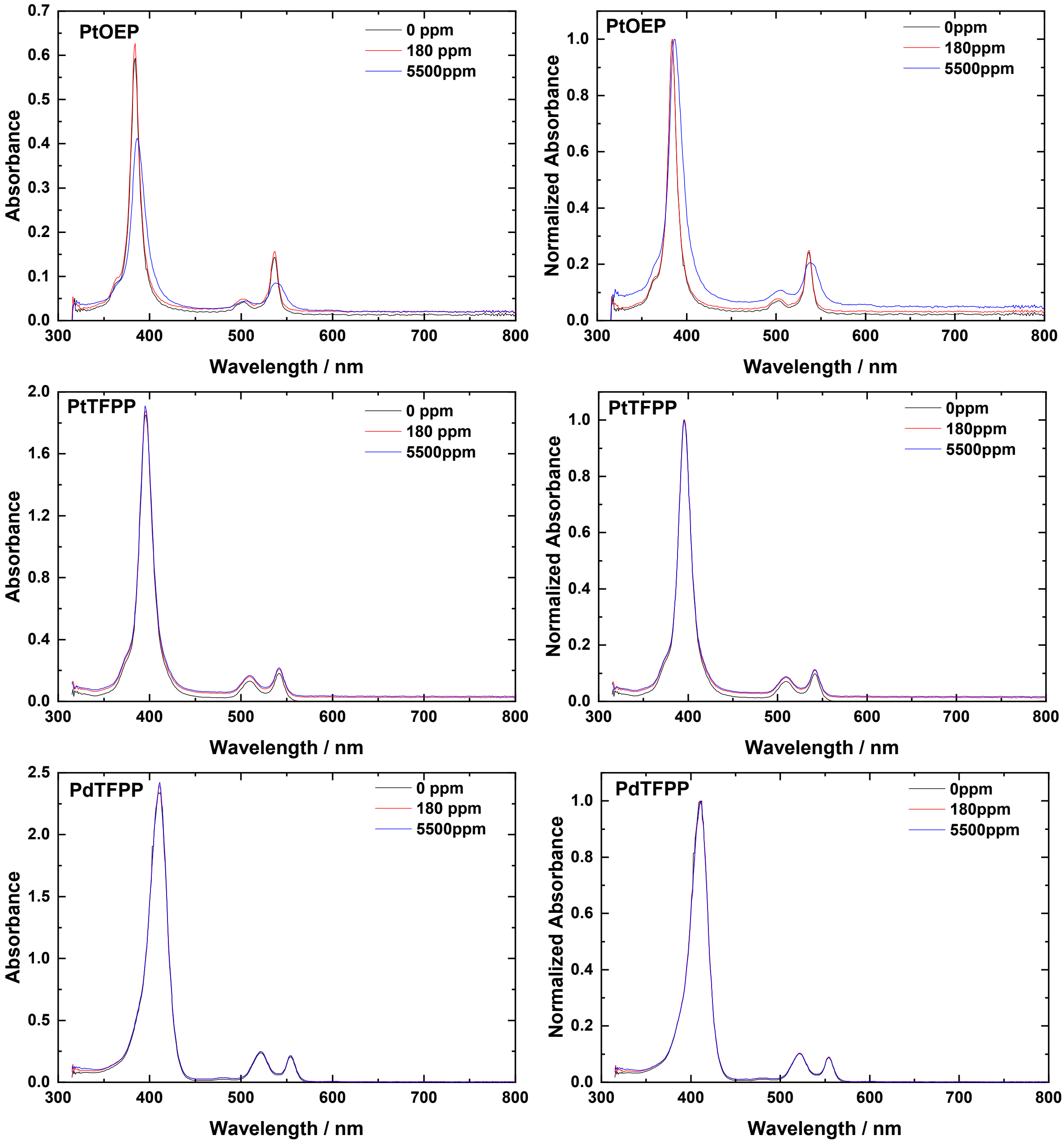

Figure S4. Absorption spectra of PS foils with immobilized porphyrins before exposure to NO_2, after exposure to 180 ppm NO_2 for 10 minutes, and after exposure to 5500 ppm NO_2 for 30 minutes. ***The right row*** *shows absorption spectra normalized to the most intence absorpiton band.*

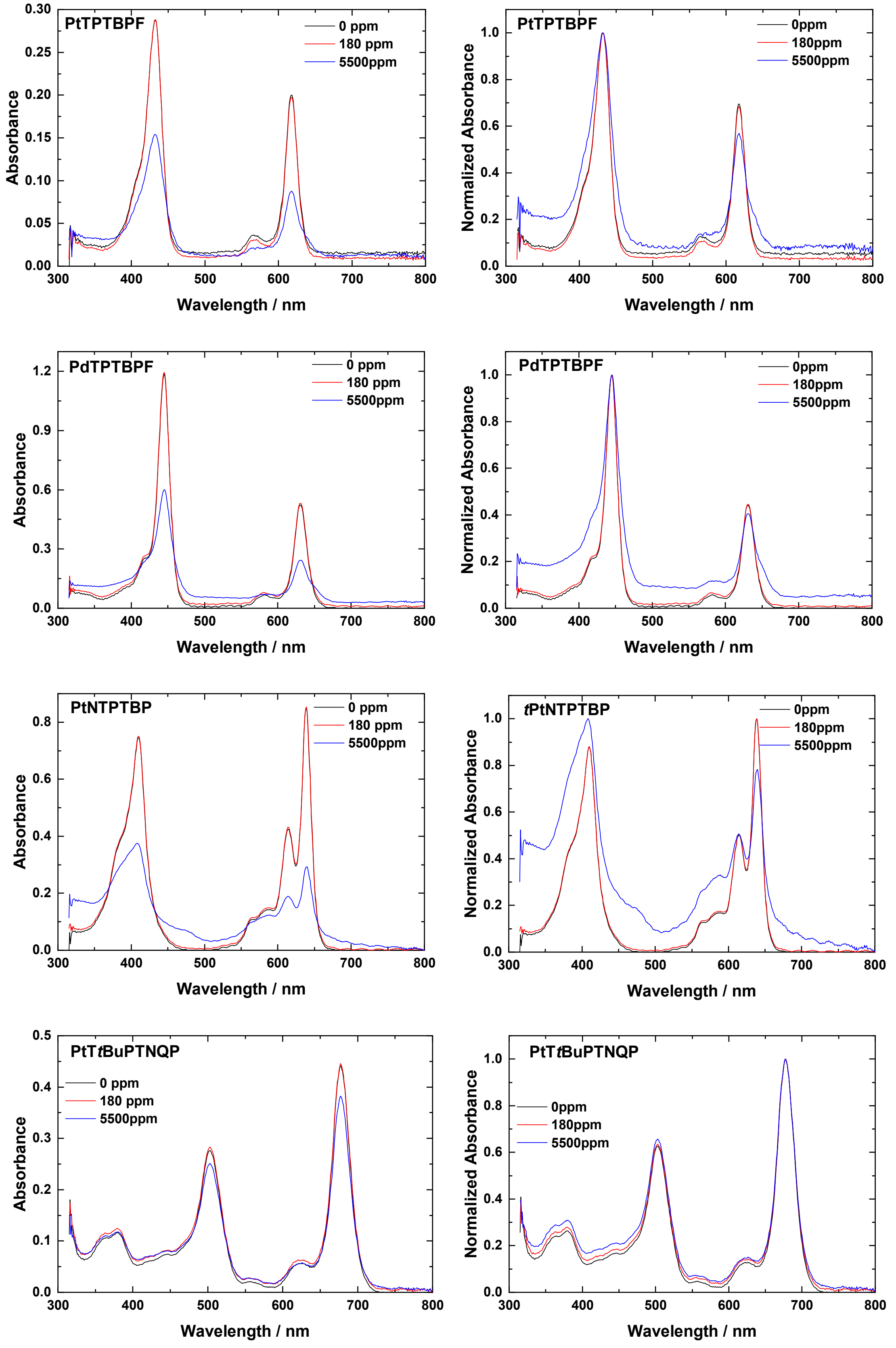

Figure S5. Absorption spectra of PS foils with immobilized π-extended porphyrins before exposure to NO_2, after exposure to 180 ppm NO_2 for 10 minutes, and after exposure to 5500 ppm NO_2 for 30 minutes. ***The right row*** *shows absorption spectra normalized to the most intence absorpiton band.*

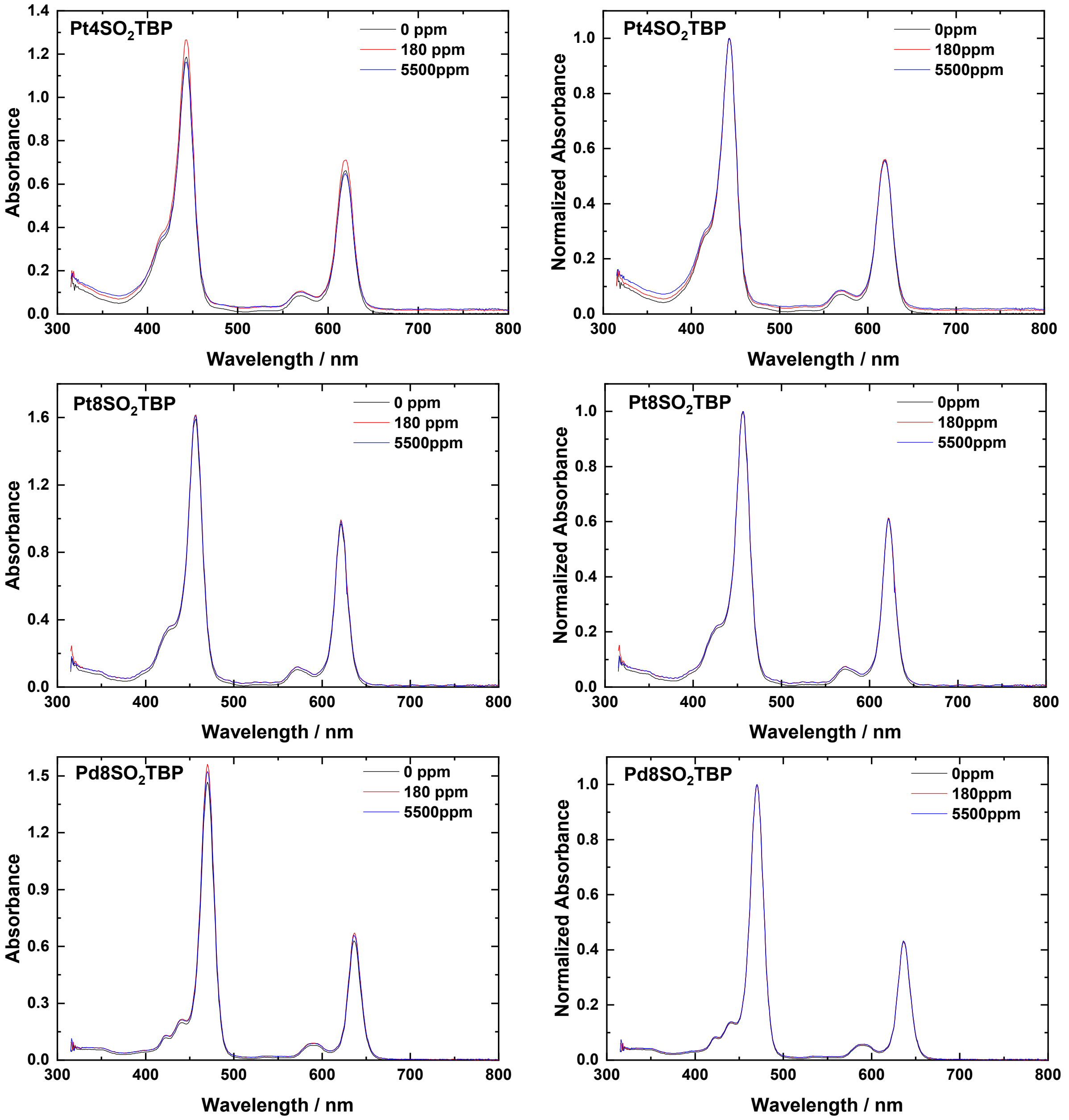

Figure S6. Absorption spectra of PS foils with immobilized π-extended porphyrins before exposure to NO_2, after exposure to 180 ppm NO_2 for 10 minutes, and after exposure to 5500 ppm NO_2 for 30 minutes. ***The right row*** *shows absorption spectra normalized to the most intence absorpiton band.*

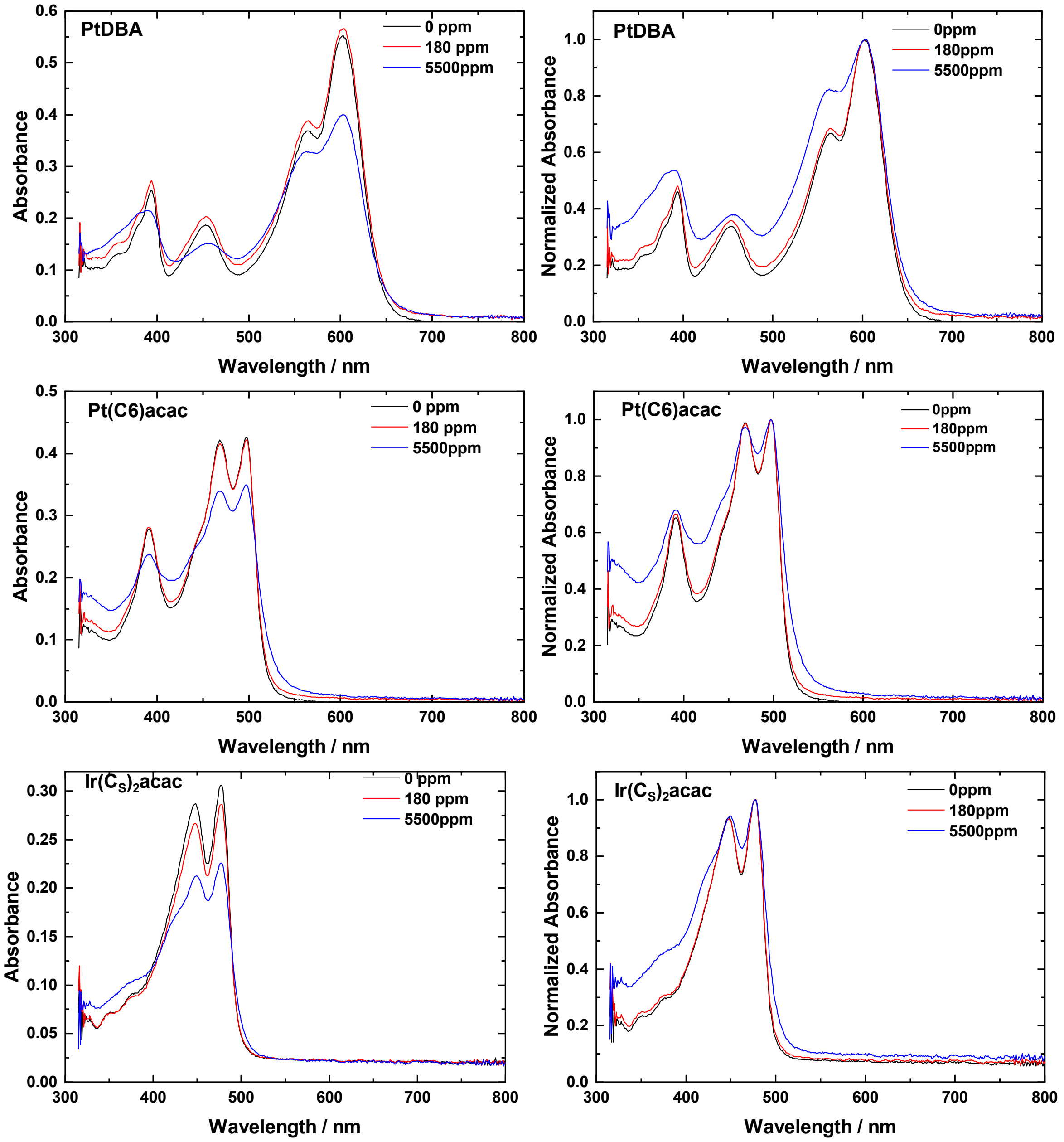

Figure S7. Absorption spectra of PS foils doped with various phosphorescent complexes before exposure to NO_2, after exposure to 180 ppm NO_2 for 10 minutes, and after exposure to 5500 ppm NO_2 for 30 minutes. ***The right row*** *shows absorption spectra normalized to the most intence absorpiton band.*

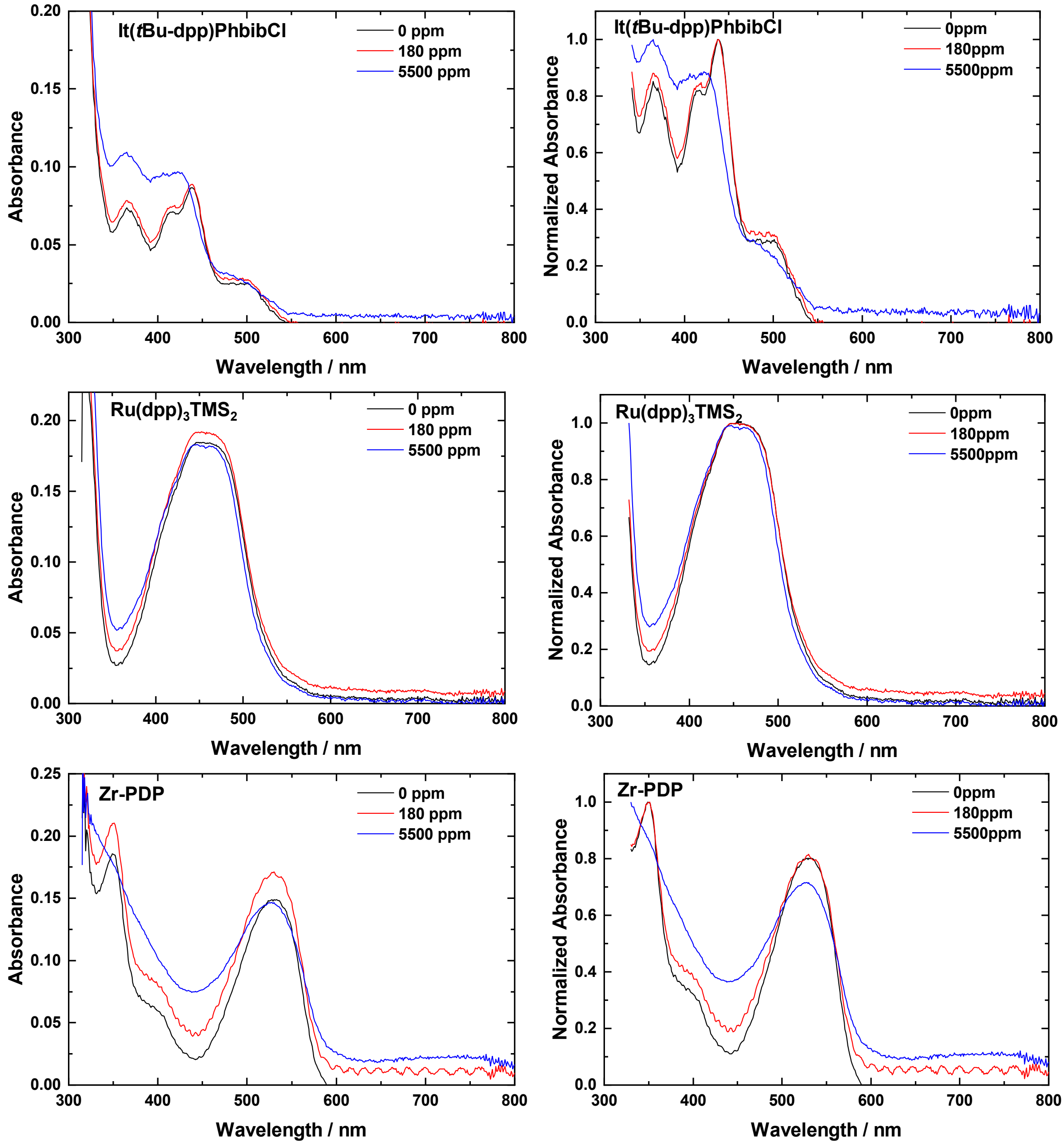

Figure S8. Absorption spectra of PS foils doped with various phosphorescent complexes before exposure to NO_2, after exposure to 180 ppm NO_2 for 10 minutes, and after exposure to 5500 ppm NO_2 for 30 minutes. ***The right row*** *shows absorption spectra normalized to the most intence absorpiton band.*

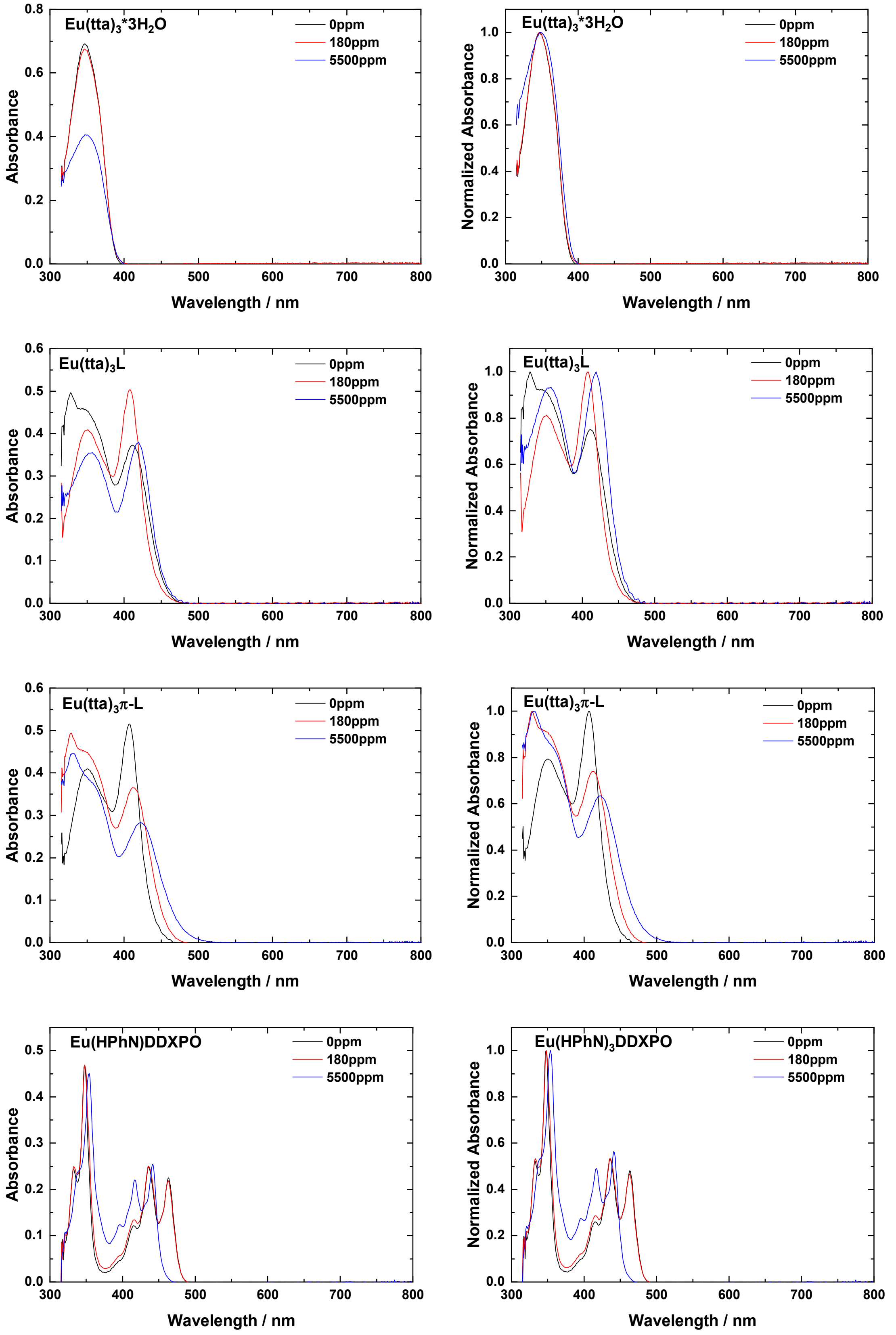

Figure S9. Absorption spectra of PS foils doped with europium complexes before exposure to NO_2, after exposure to 180 ppm NO_2 for 10 minutes, and after exposure to 5500 ppm NO_2 for 30 minutes. ***The right row*** *shows absorption spectra normalized to the most intence absorpiton band.*

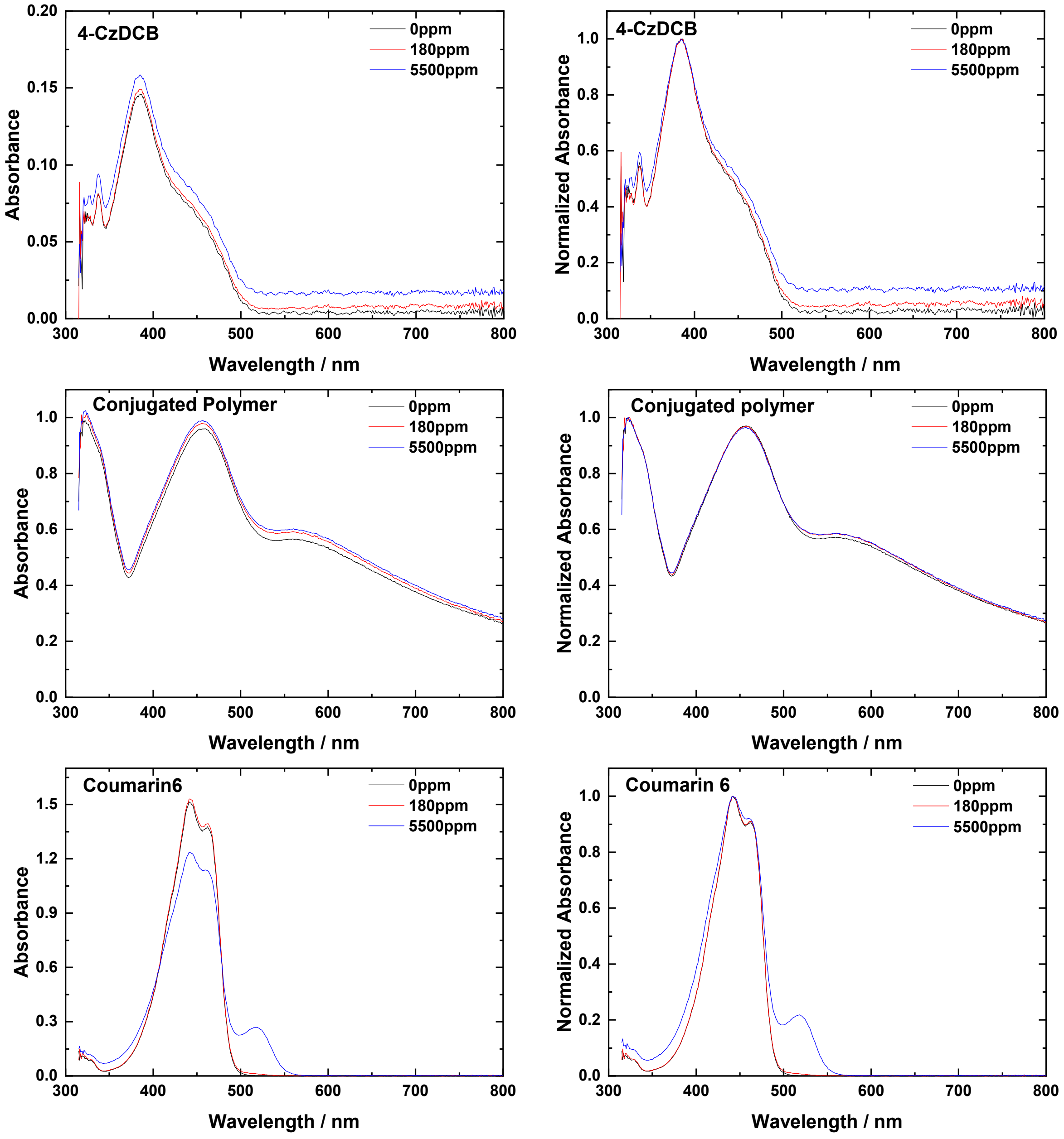

Figure S10. Absorption spectra of PS foils doped with fluorescent dyes before exposure to NO_2, after exposure to 180 ppm NO_2 for 10 minutes, and after exposure to 5500 ppm NO_2 for 30 minutes. ***The right row*** *shows absorption spectra normalized to the most intence absorpiton band.*

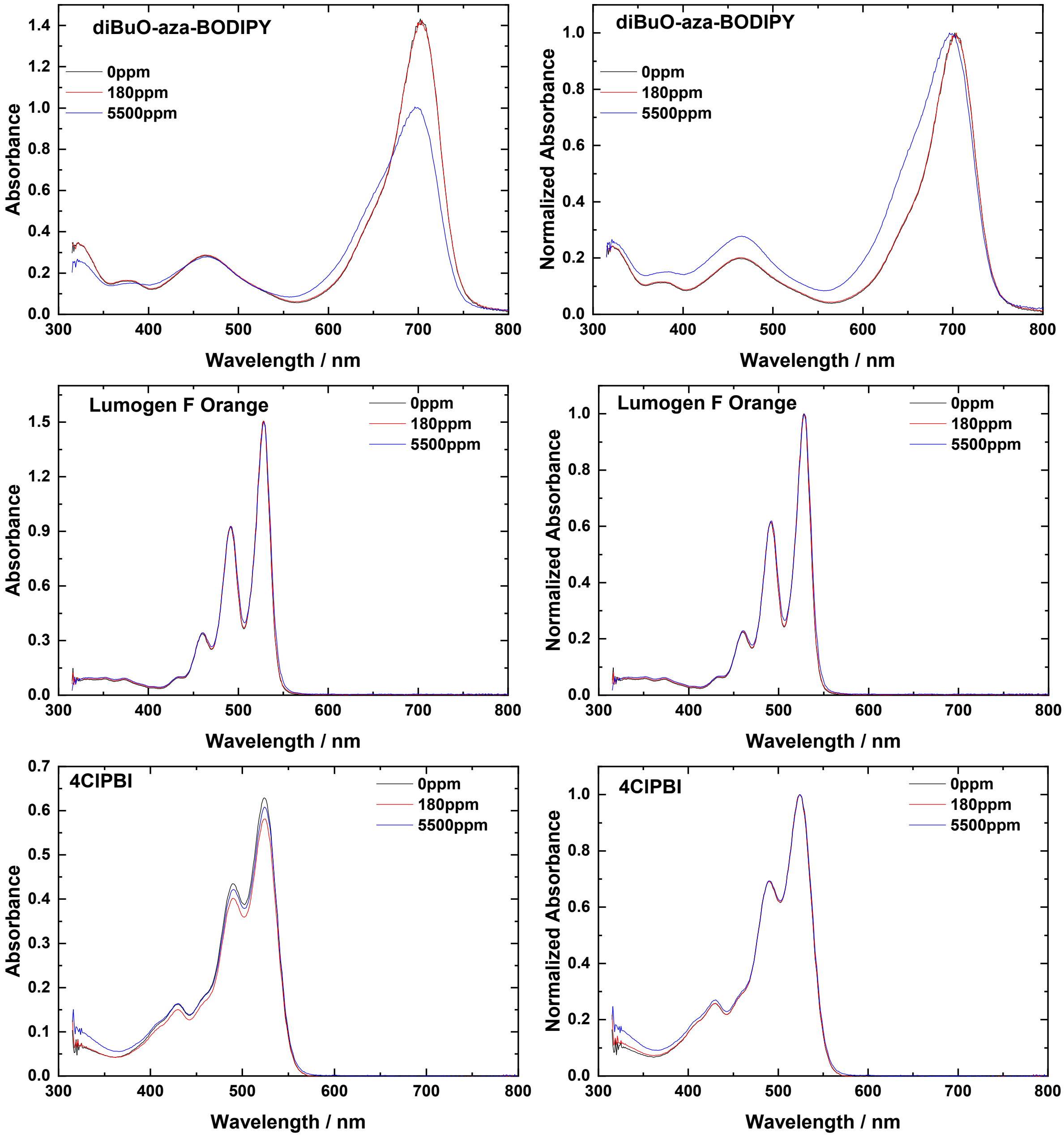

Figure S11. Absorption spectra of PS foils doped with fluorescent dyes before exposure to NO_2, after exposure to 180 ppm NO_2 for 10 minutes, and after exposure to 5500 ppm NO_2 for 30 minutes. ***The right row*** *shows absorption spectra normalized to the most intence absorpiton band.*

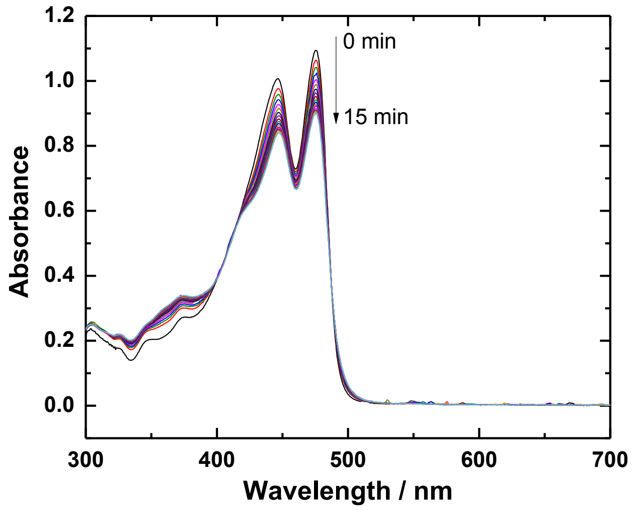

Figure S12. Absorption spectra of Ir(Cs)₂acac dissolved in toluene (C ~ 1.4· 10^{-5} mol·L^{-1}) before (0 min) and after (1- 15 min) exposure to NO_2 (C ~ 1.5 · 10^{-4} mol·L^{-1}).

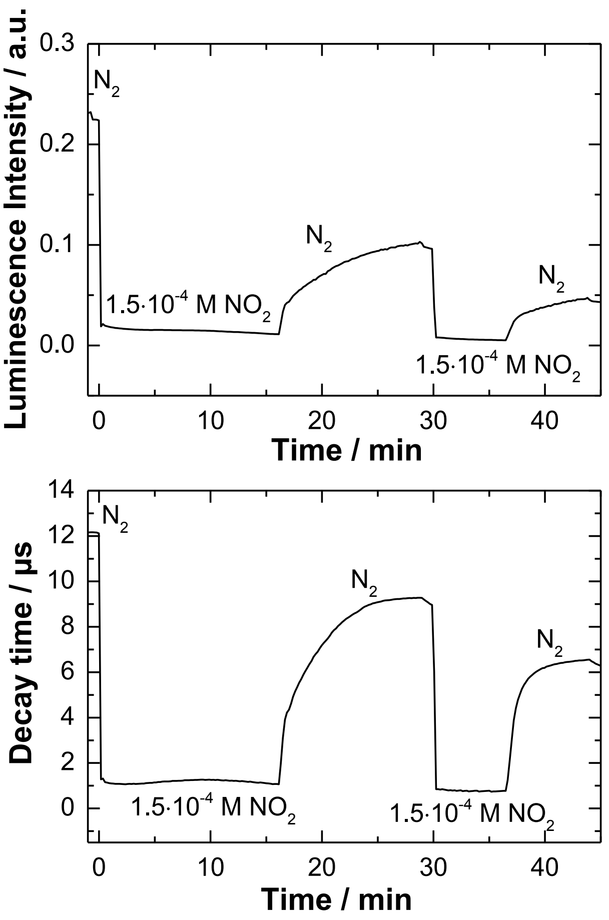

Figure S13. Response of luminescence intensity (above) and decay time (below) of Ir(Cs)₂acac in toluene (C ~ 1.4· 10^{-5} mol·L^{-1}) to NO_2.

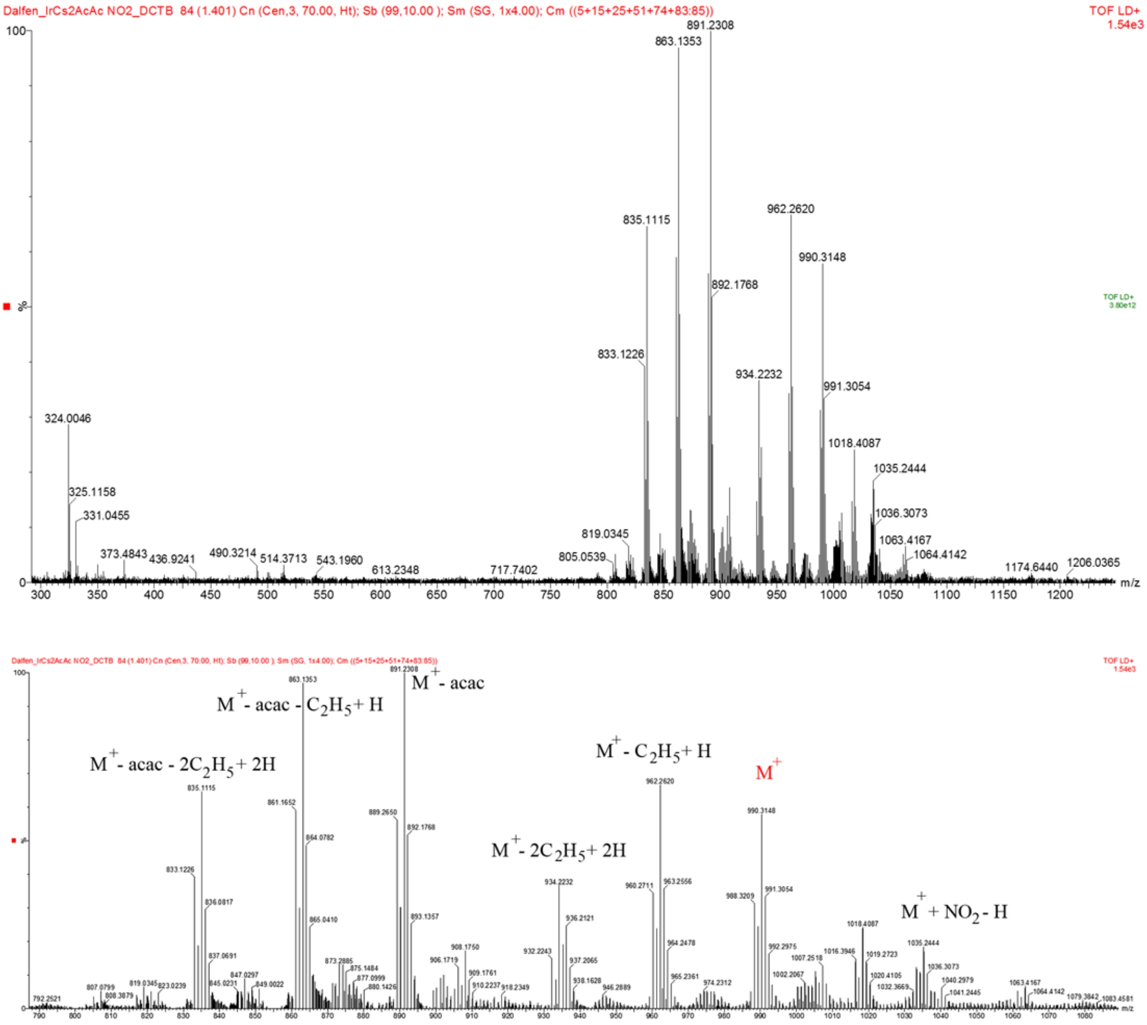

Figure S14. Mass spectra (MALDI-TOF) (matrix DCTB) of Ir(Cs)$_2$acac after exposure of toluene solutions to NO_2 (C ~ 1· 10^{-5} mol·L^{-1}). The lower part is the zoom-in of the area with main peaks.

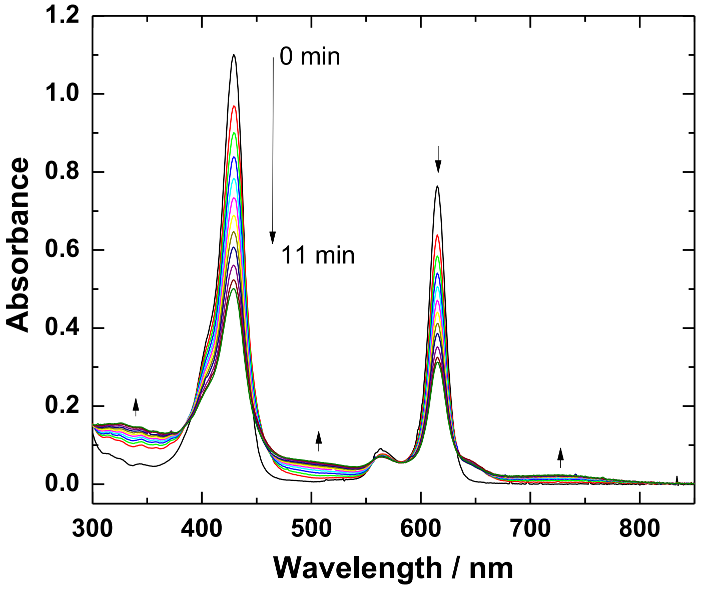

Figure S15. Absorption spectra of PtTPTBPF dissolved in toluene (C ~ 5· 10^{-6} mol·L^{-1}) before (0 min) and after (1- 11 min) exposure to NO_2 (C ~ 2.2· 10^{-4} mol·L^{-1}).

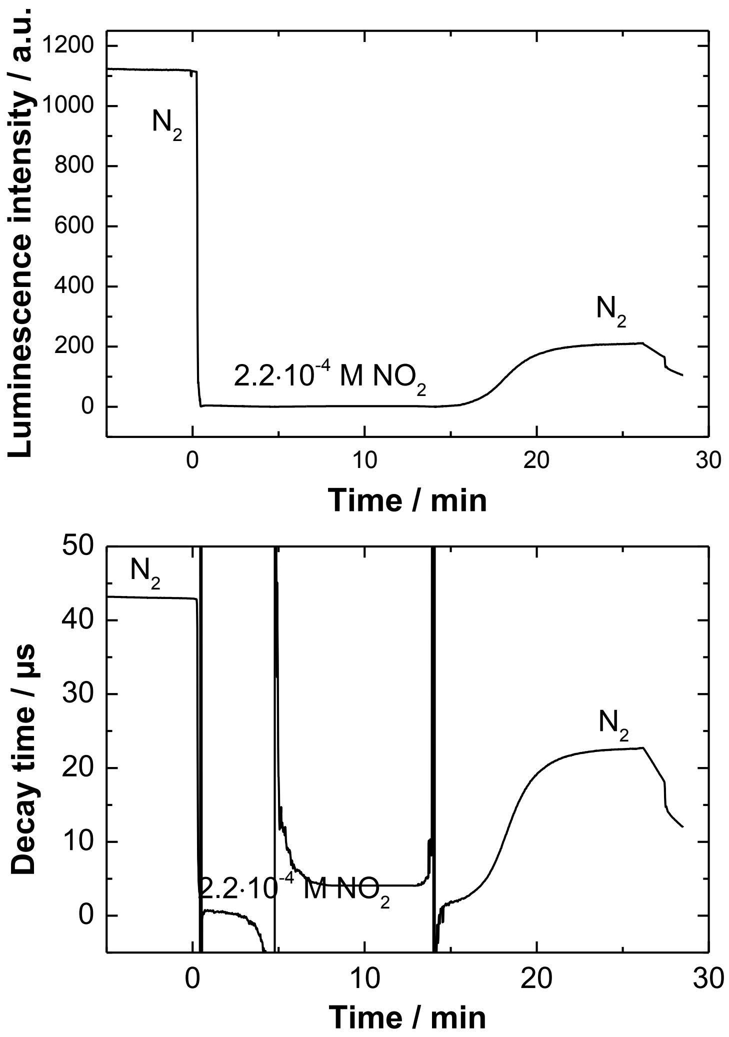

Figure S16. Response of luminescence intensity (above) and decay time (below) of PtTPTBPF in toluene (C ~ 5·10^-6^ mol·L^-1^) to NO_2. Note that due to extremely low luminescence intensity in presence of NO_2 reliable measurement of the luminescecne decay time is not possible.

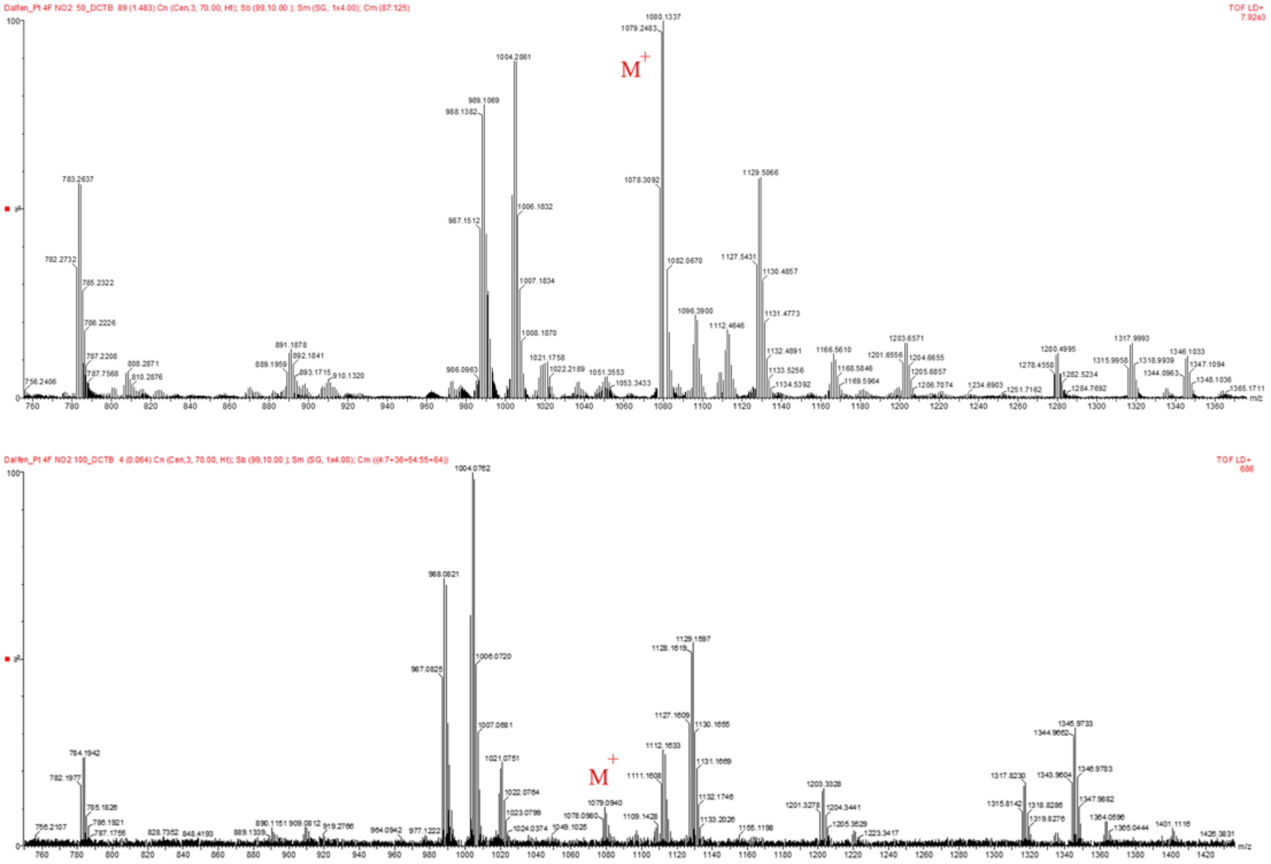

Figure S17. Mass spectra (MALDI-TOF) (matrix DCTB) of PtTPTBPF after exposure of toluene solutions to NO_2 ($C \sim 2.2 \cdot 10^{-4}$ mol·L^{-1}, upper part; $C \sim 4.4 \cdot 10^{-4}$ mol·L^{-1}, lower part).

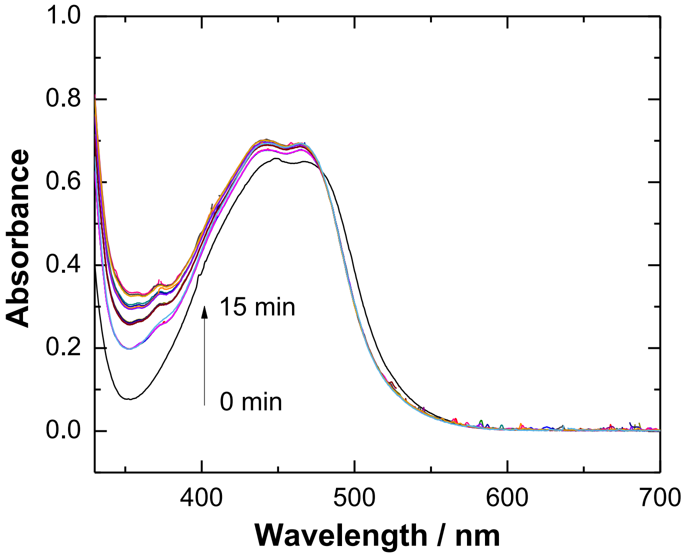

Figure S18. Absorption spectra of $Ru(dpp)_3(TMS)_2$ dissolved in toluene ($C \sim 2 \cdot 10^{-5}$ mol·L^{-1}) before (0 min) and after (1- 11 min) exposure to NO_2 ($C \sim 6 \cdot 10^{-4}$ mol·L^{-1}added in 4 portions).

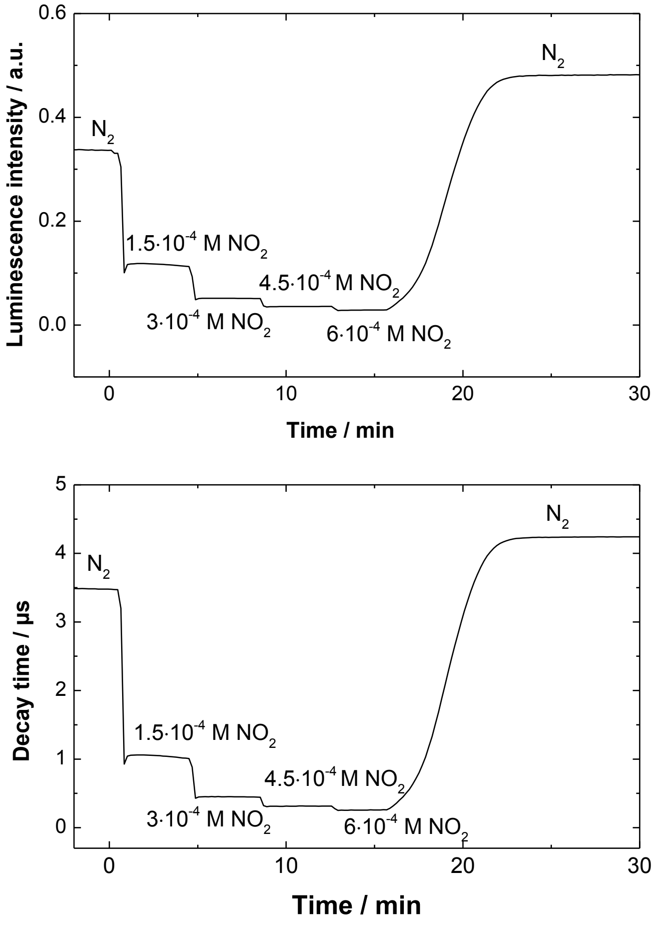

Figure S19. Response of luminescence intensity (above) and decay time (below) of Ru(dpp)$_3$(TMS)$_2$ in toluene (C ~ 2 · 10^{-5} mol·L^{-1}) to NO$_2$.

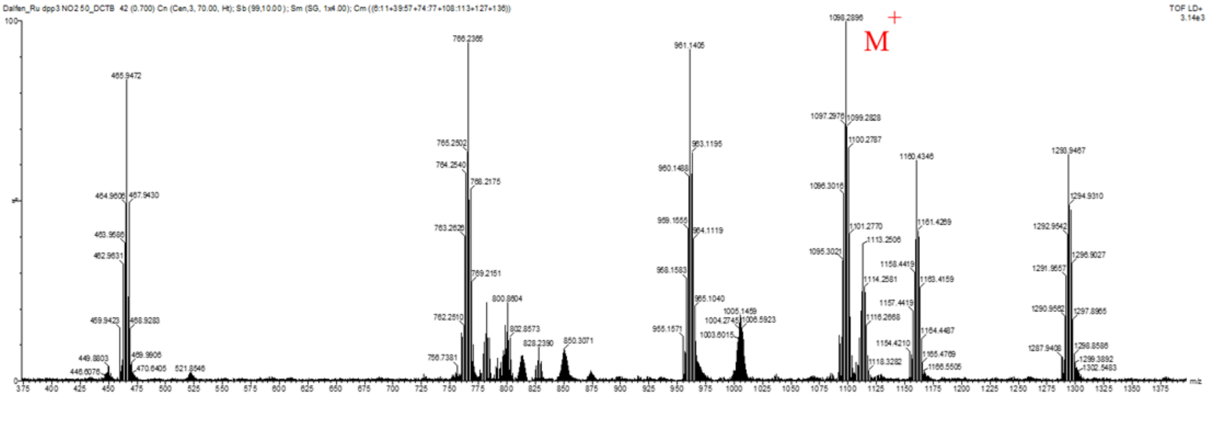

Figure S20. Mass spectrum (MALDI-TOF) (matrix DCTB) of Ru(dpp)$_3$(TMS)$_2$ after exposure of toluene solution to NO$_2$.

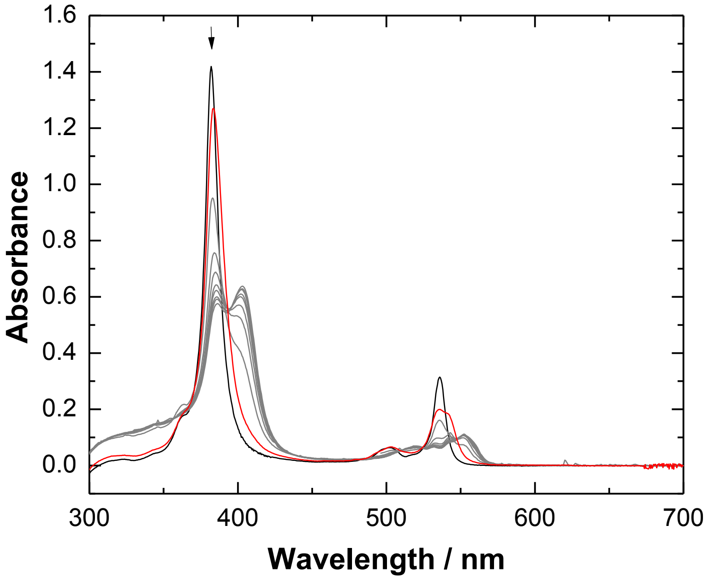

Figure S21. Absorption spectra of PtOEP dissolved in toluene (C ~ 8· 10^{-6} mol·L^{-1}) before (black line) and after (1- 9 min) exposure to NO_2 (C ~ 1.5· 10^{-4} mol·L^{-1}), gray lines. Red line shows absorption spectrum after NO_2 was removed by bubbling nitrogen through the solution for 15 min.

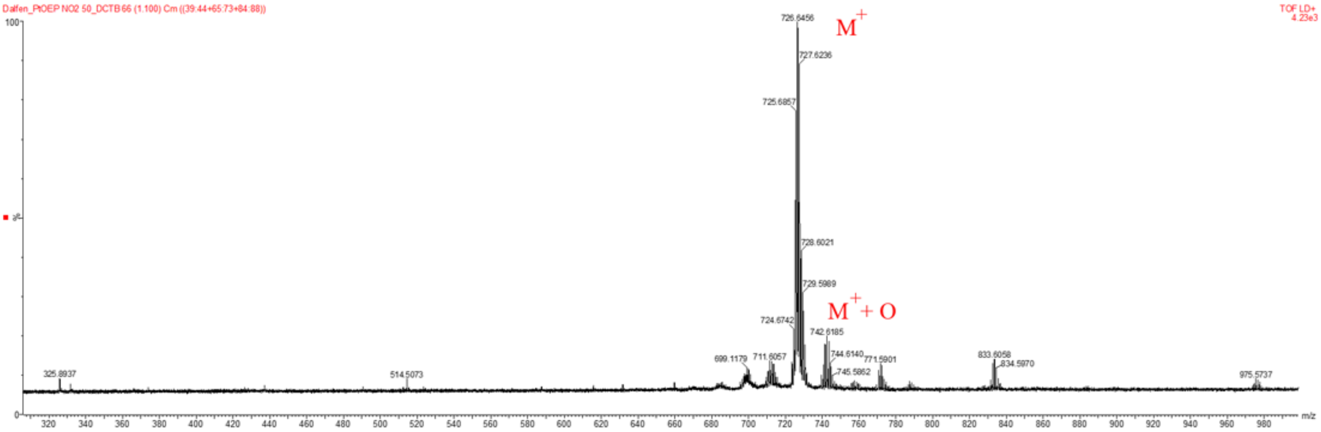

Figure S22. Mass spectrum (MALDI-TOF) (matrix DCTB) of PtOEP after exposure of toluene solution to NO_2.

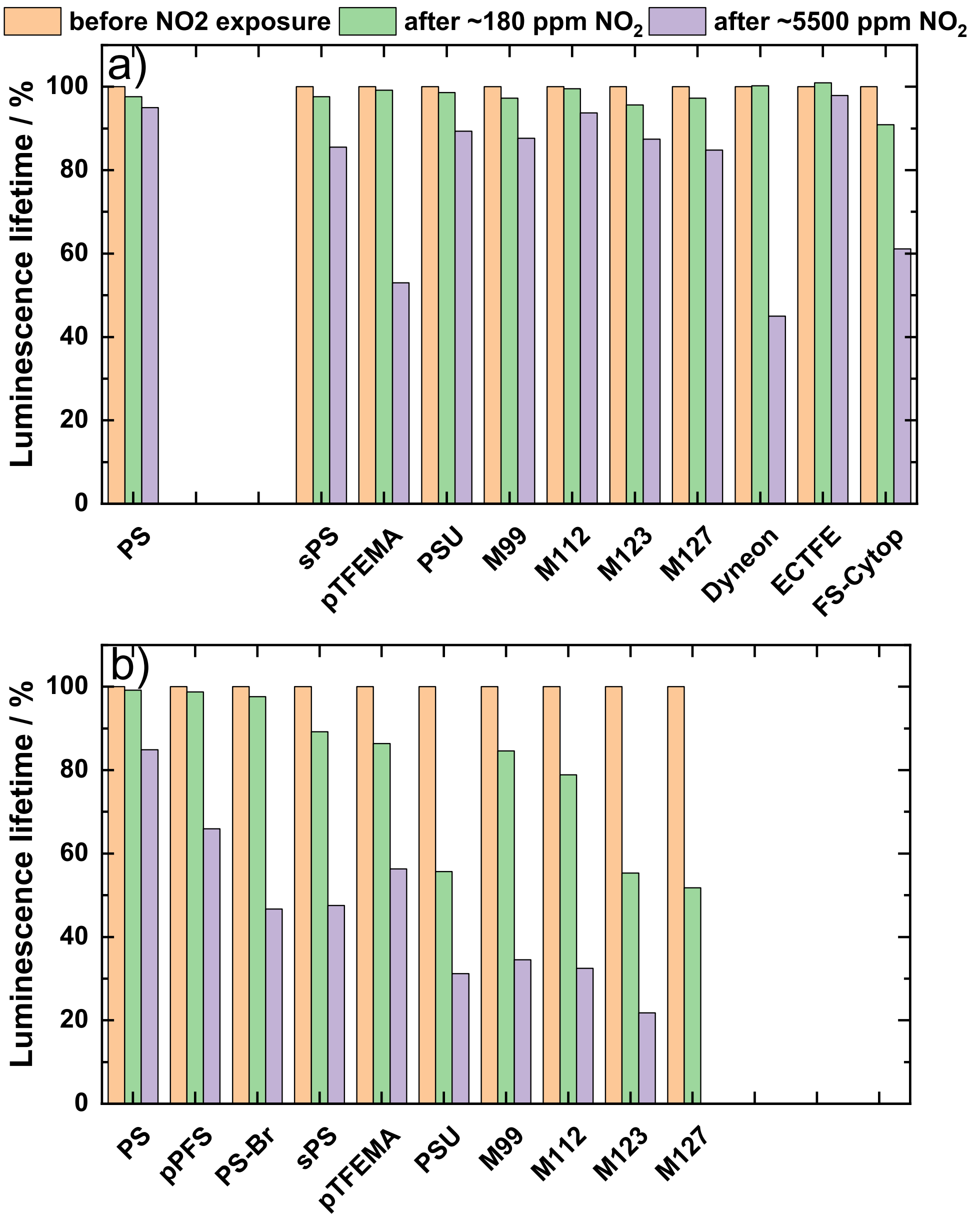

Figure S23. Comparison of normalized luminescence lifetime of sensor foils of (a) PtTFPP and (b) PtTPTBPF in different polymers, before exposure to NO_2, after exposure to 180 ppm NO_2, and after exposure to 5500 ppm NO_2. Time of exposure was about 15 minutes in all cases.

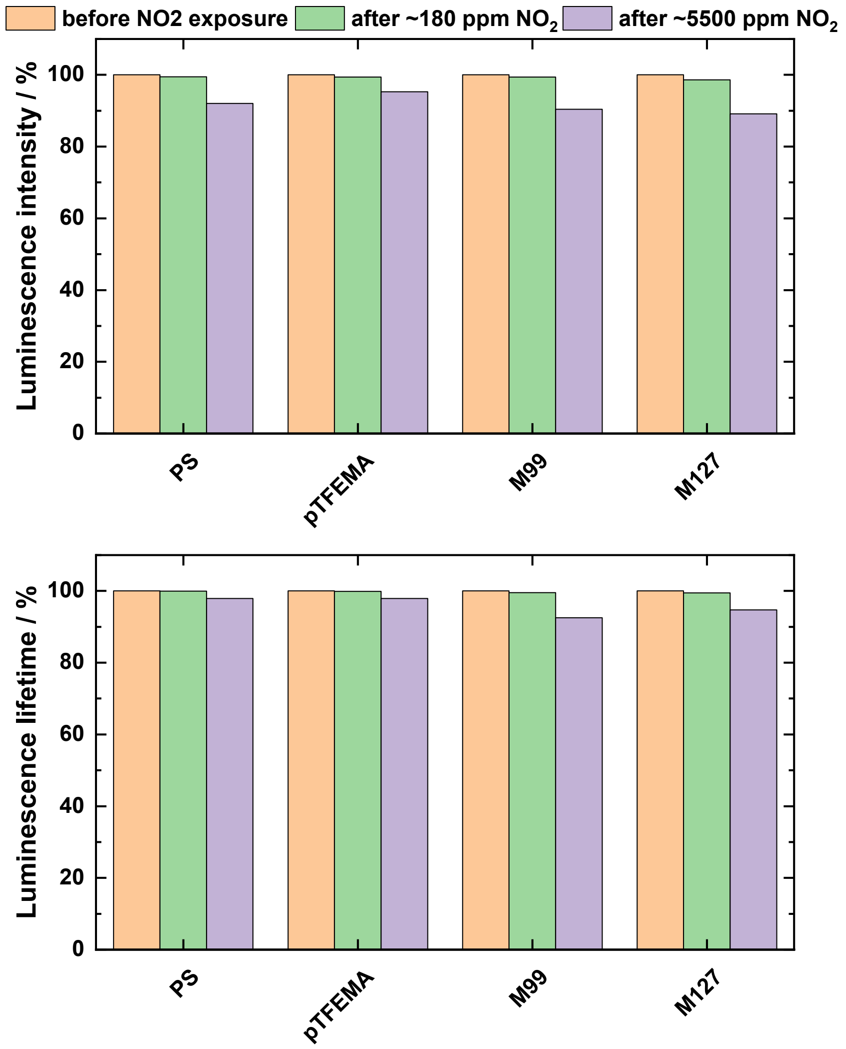

Figure S24. Comparison of normalized luminescence intensity (top) and lifetime (bottom) of sensor foils of Pt8SO$_2$TPTBP in different polymers, before exposure to NO$_2$, after exposure to 180 ppm NO$_2$, and after exposure to 5500 ppm NO$_2$. Time of exposure was about 15 minutes in all cases.

Table S5. Fit parameters for the decay time-based Stern-Volmer plots (fit with eq.3) obtained for immobilized Pt8SO$_2$TPTBP

Polymer	Quencher					
	NO_2			O_2		
	K_{SV1}, hPa^{-1}	f	R^2	K_{SV1}, hPa^{-1}	f	R^2
PS	0.34	0.65	0.996	0.020	0.86	0.999
M99	0.42	0.50	0.987	0.011	0.90	0.999
pTFEMA	0.46	0.85	0.999	0.035	0.94	0.999
M127	0.74	0.81	0.999	0.039	0.90	0.999

References

(1) Jankova, K.; Hvilsted, S. Preparation of Poly(2,3,4,5,6-Pentafluorostyrene) and Block Copolymers with Styrene by ATRP. *Macromolecules* **2003**, *36* (5), 1753–1758. https://doi.org/10.1021/ma021039m.

(2) Hutter, L. H.; Müller, B. J.; Koren, K.; Borisov, S. M.; Klimant, I. Robust Optical Oxygen Sensors Based on Polymer-Bound NIR-Emitting Platinum(II)–Benzoporphyrins. *J. Mater. Chem. C* **2014**, *2* (36), 7589–7598. https://doi.org/10.1039/C4TC00983E.

(3) Nacht, B.; Larndorfer, C.; Sax, S.; Borisov, S. M.; Hajnsek, M.; Sinner, F.; List-Kratochvil, E. J. W.; Klimant, I. Integrated Catheter System for Continuous Glucose Measurement and Simultaneous Insulin Infusion. *Biosensors and Bioelectronics* **2015**, *64*, 102–110. https://doi.org/10.1016/j.bios.2014.08.012.

(4) Zach, P. W.; Freunberger, S. A.; Klimant, I.; Borisov, S. M. Electron-Deficient Near-Infrared Pt(II) and Pd(II) Benzoporphyrins with Dual Phosphorescence and Unusually Efficient Thermally Activated Delayed Fluorescence: First Demonstration of Simultaneous Oxygen and Temperature Sensing with a Single Emitter. *ACS Appl. Mater. Interfaces* **2017**, *9* (43), 38008–38023. https://doi.org/10.1021/acsami.7b10669.

(5) Banala, S.; Wurst, K.; Kräutler, B. Panchromatic π-Extended Porphyrins from Conjugation with Quinones. *ChemPlusChem* **2016**, *81* (5), 477–488. https://doi.org/10.1002/cplu.201600115.

(6) Borisov, S. M.; Klimant, I. Ultrabright Oxygen Optodes Based on Cyclometalated Iridium(III) Coumarin Complexes. *Anal. Chem.* **2007**, *79* (19), 7501–7509. https://doi.org/10.1021/ac0710836.

(7) Shafikov, M. Z.; Daniels, R.; Kozhevnikov, V. N. Unusually Fast Phosphorescence from Ir(III) Complexes via Dinuclear Molecular Design. *J. Phys. Chem. Lett.* **2019**, *10* (22), 7015–7024. https://doi.org/10.1021/acs.jpclett.9b03002.

(8) Borisov, S. M.; Klimant, I. Efficient Metallation in Diphenylether – A Convenient Route to Luminescent Platinum(II) Complexes. *Dyes and Pigments* **2009**, *83* (3), 312–316. https://doi.org/10.1016/j.dyepig.2009.05.008.

(9) Borisov, S. M.; Saf, R.; Fischer, R.; Klimant, I. Synthesis and Properties of New Phosphorescent Red Light-Excitable Platinum(II) and Palladium(II) Complexes with Schiff Bases for Oxygen Sensing and Triplet–Triplet Annihilation-Based Upconversion. *Inorg. Chem.* **2013**, *52* (3), 1206–1216. https://doi.org/10.1021/ic301440k.

(10) Klimant, Ingo.; Wolfbeis, O. S. Oxygen-Sensitive Luminescent Materials Based on Silicone-Soluble Ruthenium Diimine Complexes. *Anal. Chem.* **1995**, *67* (18), 3160–3166. https://doi.org/10.1021/ac00114a010.

(11) Zhang, Y.; Lee, T. S.; Favale, J. M.; Leary, D. C.; Petersen, J. L.; Scholes, G. D.; Castellano, F. N.; Milsmann, C. Delayed Fluorescence from a Zirconium(Iv) Photosensitizer with Ligand-to-Metal Charge-Transfer Excited States. *Nat. Chem.* **2020**, *12* (4), 345–352. https://doi.org/10.1038/s41557-020-0430-7.

(12) Steinegger, A.; Klimant, I.; Borisov, S. M. Purely Organic Dyes with Thermally Activated Delayed Fluorescence-A Versatile Class of Indicators for Optical Temperature Sensing. *Advanced Optical Materials* **2017**, 1700372. https://doi.org/10.1002/adom.201700372.

(13) Borisov, S. M.; Fischer, R.; Saf, R.; Klimant, I. Exceptional Oxygen Sensing Properties of New Blue Light-Excitable Highly Luminescent Europium(III) and Gadolinium(III) Complexes. *Adv. Funct. Mater.* **2014**, *24* (41), 6548–6560. https://doi.org/10.1002/adfm.201401754.

(14) Yang, C.; Fu, L.-M.; Wang, Y.; Zhang, J.-P.; Wong, W.-T.; Ai, X.-C.; Qiao, Y.-F.; Zou, B.-S.; Gui, L.-L. A Highly Luminescent Europium Complex Showing Visible-Light-Sensitized Red Emission: Direct Observation of the Singlet Pathway. *Angew. Chem. Int. Ed.* **2004**, *43* (38), 5010–5013. https://doi.org/10.1002/anie.200454141.

(15) Borisov, S. M.; Klimant, I. Blue LED Excitable Temperature Sensors Based on a New Europium(III) Chelate. *J Fluoresc* **2008**, *18* (2), 581–589. https://doi.org/10.1007/s10895-007-0302-1.
(16) Herguth, P.; Jiang, X.; Liu, M. S.; Jen, A. K.-Y. Highly Efficient Fluorene- and Benzothiadiazole-Based Conjugated Copolymers for Polymer Light-Emitting Diodes. *Macromolecules* **2002**, *35* (16), 6094–6100. https://doi.org/10.1021/ma020405z.
(17) Strobl, M.; Rappitsch, T.; Borisov, S. M.; Mayr, T.; Klimant, I. NIR-Emitting Aza-BODIPY Dyes – New Building Blocks for Broad-Range Optical PH Sensors. *Analyst* **2015**, *140* (21), 7150–7153. https://doi.org/10.1039/C5AN01389E.
(18) Seybold, G. New Perylene and Violanthrone Dyestuffs for Fluorescent Collectors. *Dyes and Pigments* **1989**, *11* (4), 303–317. https://doi.org/10.1016/0143-7208(89)85048-X.
(19) Borisov, S. M.; Lehner, P.; Klimant, I. Novel Optical Trace Oxygen Sensors Based on Platinum(II) and Palladium(II) Complexes with 5,10,15,20-Meso-Tetrakis-(2,3,4,5,6-Pentafluorphenyl)-Porphyrin Covalently Immobilized on Silica-Gel Particles. *Anal. Chim. Acta* **2011**, *690* (1), 108–115. https://doi.org/10.1016/j.aca.2011.01.057.
(20) Liu, B.; Hu, W.; Zhao, S.; Chen, C.; Wu, Z.; Matsumoto, T. Methylated and Trifluoromethylated Poly(Aryl Ethers). *Polym J* **2003**, *35* (8), 628–633. https://doi.org/10.1295/polymj.35.628.
